# Supplementary material for: Personal ethical settings for driverless cars and the utility paradox: An ethical analysis of public attitudes in UK and Japan
Source: PLoS One. 2022 Nov 15;17(11):e0275812. doi: 10.1371/journal.pone.0275812 (PMC9665398; doi:10.1371/journal.pone.0275812)
Supplement: S1 File — (DOCX) [file pone.0275812.s001.docx]

**Supporting information**

**S1 Table. Demographic information of UK and Japanese participants**

**
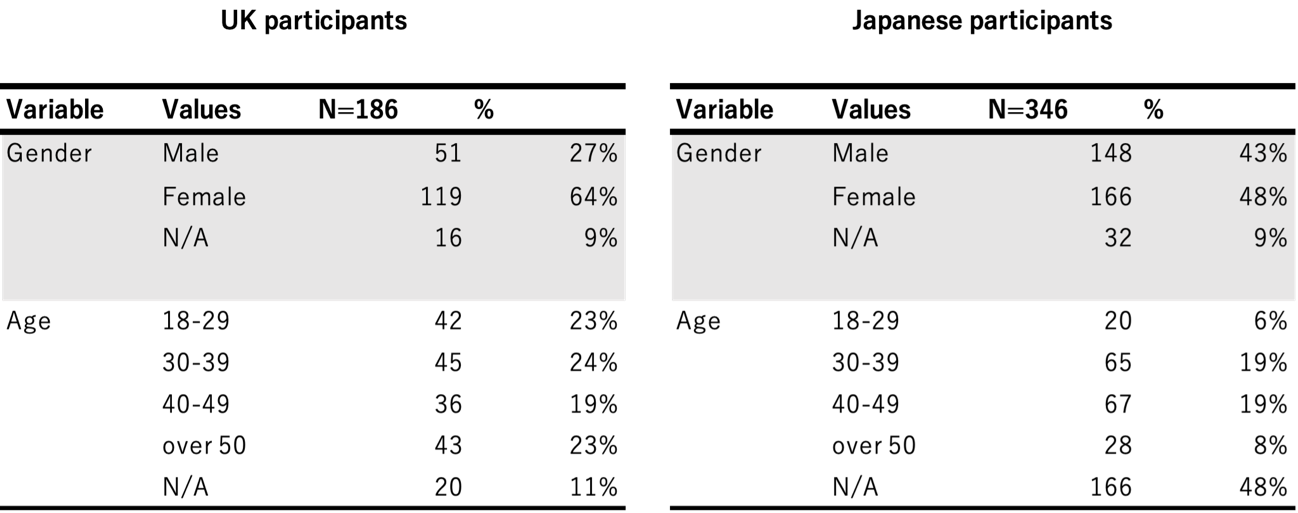
**

**S2 Table. Comparison of hyperparameter estimates**

|  | **UK Sample** | | **Japanese Sample** | |
| --- | --- | --- | --- | --- |
| **Parameter** | **Mean** | **HDI** | **Mean** | **HDI** |
| **Pedestrian** | -10.70 | [-13.30, -8.14] | 4.18 | [1.95, 6.42] |
| **Occupant** | -3.17 | [-5.64, -0.73] | -6.47 | [-8.70, -4.25] |
| **Tau** | 0.291 | [0.237, 0.348] | 0.388 | [0.313, 0.467] |
| **t** | 0.0034 | [0.0025, 0.0044] | 0.0045 | [0.0033, 0.0056] |
| **t2** | 25.18 | [15.28, 35.95] | 17.39 | [9.82, 25.63] |

**S1 Fig. Survey flow**

**
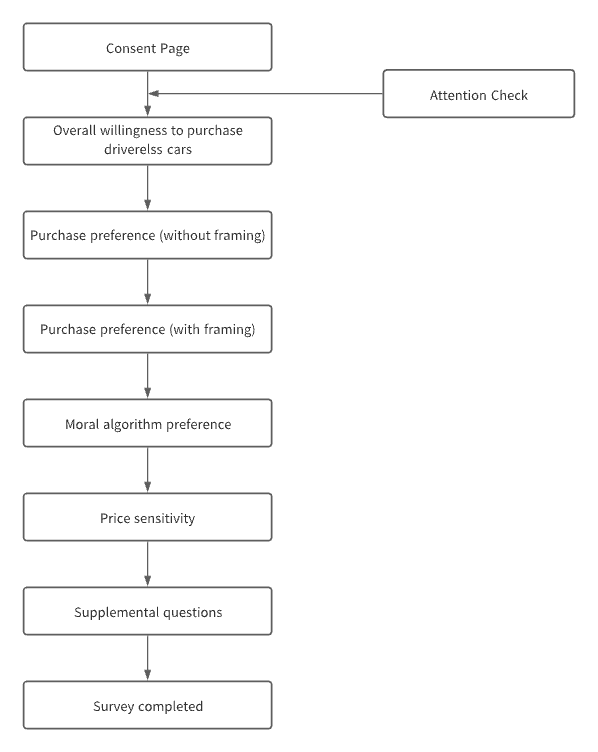
**

**S2 Fig. Model fit**

| 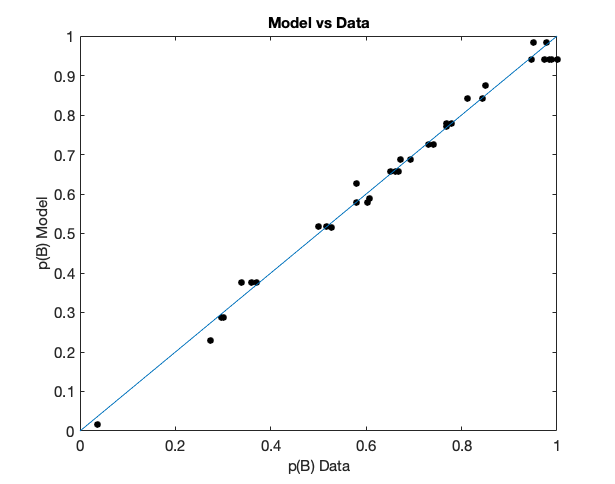 |
| --- |
| **S2-1 Fig.** Plot of average proportion of UK participants choosing option B (x-axis) against the mean of the posterior probabilities for each participant (y-axis). Points lying on the diagonal indicate perfect agreement between data and model when averaged over participants. |

| 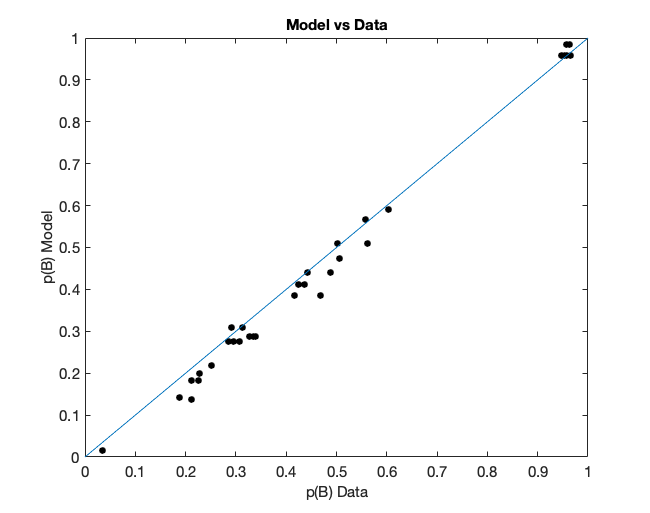 |
| --- |
| **S2-2 Fig.** Plot of average proportion of Japanese participants choosing option B (x-axis) against the mean of the posterior probabilities for each participant (y-axis). Points lying on the diagonal indicate perfect agreement between data and model when averaged over participants. |

**S3 Fig. Histogram of mean preference strength values of “Save the Most” versus “Save the Pedestrians” (left side) and “Save the Most” versus “Save the Occupants” (right side).**

**S4 Text. Survey text (English and Japanese)**

**Supporting information**

**S4 Text. Survey text (English and Japanese)**

**Material 1: UK questionnaire**

Start of Block: Informed Consent

Q1 **General Information**   **The aim of this study is to investigate how people think about driverless cars, and in particular how they should be programmed to respond in the event of a collision. The findings will be published in a peer-reviewed journal and also included in a dissertation by a student involved in the project. Please read through this information carefully before agreeing to participate by ticking the ‘yes’ box below. You may ask any questions before deciding to take part by contacting the researcher (details below).**   **The survey takes about 12 minutes. This is not a test of your knowledge, and no background knowledge is required. You will be asked about different options for programming driverless cars and asked to indicate your preference. You will be required to answer most of the questions, though for any personal questions you will have an option to indicate you prefer not to say. Reimbursement for the survey will be paid at a rate of £7.50/hour (£1.25 for 10-minute survey).**     **Do I have to take part?** Please note that your participation is voluntary. If you do decide to take part, you may withdraw at any point during the questionnaire for any reason before submitting your answers by pressing the ‘Exit’ button/closing the browser. However, we are only able to reimburse participants who complete the full survey.   **How will my data be used?** Your answers will be completely anonymous, and we will take all reasonable measures to ensure that they remain confidential. Your data will be stored in a password-protected file and may be used in academic publications. Your IP address will not be stored. Research data will be stored for a minimum of three years after publication or public release. The data that we collect from you may be transferred to, stored and/or processed at a destination outside the UK and the European Economic Area ("EEA"). By submitting your personal data, you agree to this transfer, storing, or processing.      **Who will have access to my data?** The University of Oxford is the data controller with respect to your personal data, and as such will determine how your personal data is used in the study. The University will process your personal data for the purpose of the research outlined above. Research is a task that we perform in the public interest.  Further information about your rights with respect to your personal data is available from https://compliance.admin.ox.ac.uk/individual-rights. Responsible members of the University of Oxford and funders may be given access to data for monitoring and/or audit of the study to ensure we are complying with guidelines, or as otherwise required by law. The lead researcher is Professor Dominic Wilkinson, who is attached to the Oxford Uehiro Centre for Practical Ethics at the University of Oxford. This project has been reviewed by, and received ethics clearance through, the University of Oxford Central University Research Ethics Committee.      **Who do I contact if I have a concern about the study or I wish to complain?** If you have a concern about any aspect of this study, please speak to Professor Dominic Wilkinson (dominic.wilkinson@philosophy.ox.ac.uk), and I will do my best to answer your query. I will acknowledge your concern within 10 working days and give you an indication of how it will be dealt with. If you remain unhappy or wish to make a formal complaint, please contact the Chair of the Research Ethics Committee at the University of Oxford who will seek to resolve the matter as soon as possible: Chair, Social Sciences & Humanities Interdivisional Research Ethics Committee; Email: ethics@socsci.ox.ac.uk; Address: Research Services, University of Oxford, Wellington Square, Oxford OX1 2JD.

 Please note that you may only participate in this survey if you are at least 18 years of age. If you agree to participate and have read the terms above, please check the relevant box below to get started.

- I agree to participate in this study. (1)

End of Block: Informed Consent

Start of Block: PROLIFIC ID

| 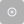 |
| --- |

Q1 Please entre your Prolific ID

________________________________________________________________

End of Block: PROLIFIC ID

Start of Block: Attention Check

Q2 It is important that you take the time to read all instructions and that you read questions carefully before you answer them. Previous research has found that some people do not take the time to read everything that is displayed in surveys. The questions below serve to test whether you actually take the time to do so. Therefore, if you read this, please answer six on the first question, divide that number by two and use the result as the answer on the second question in order to participate.

Q3 I would prefer to live in a city with many cultural opportunities, even if the cost of living were higher.

- Completely agree (1)
- 1 (2)
- 2 (3)
- 3 (4)
- 4 (5)
- 5 (6)
- 6 (7)

Q4 I would prefer to live in a large city rather than a small city.

- Don't agree at all (1)
- 1 (2)
- 2 (3)
- 3 (4)
- 4 (5)
- 5 (6)
- 6 (7)

End of Block: Attention Check

Start of Block: Sorry

Q5
**Failed Attention Check**
 
Sorry, but your previous answers indicate that you did not read the instructions and hence, we cannot be sure if you would understand the purpose and the explanations in our study. Thanks for your time.

End of Block: Sorry

Start of Block: Welcome to Study

Q6
**Welcome to our study**
  In this study, we want to know your thoughts on driverless cars and what kind of driverless car you want. 


Driverless cars will be much safer than cars driven by people. The introduction of fully autonomous vehicles (driverless cars) is predicted to reduce the number of traffic accidents by 90-94%. But in driverless cars, the driver has no opportunity to control the car in an emergency, for instance, when facing a collision. We will ask you which type of driverless car you prefer. 
 
For this survey we would like you to assume that driverless cars are safe, and function without any risk of failure, including the risk of being hacked. Moreover, for these questions please assume that there is no concern about data privacy or liability for accidents if you owned a driverless car.

| Page Break |  |
| --- | --- |

Q7 Imagine that in ten years’ time, driverless cars are the same cost and have the same features as a regular car. However, a driverless car will be much safer than a regular car. 


**How likely are you to buy a driverless car if you needed to replace your existing car?**

- Not at all likely (1)
- 2 (2)
- 3 (3)
- Neither likely nor unlikely (4)
- 5 (5)
- 6 (6)
- Extremely likely (7)

| Page Break |  |
| --- | --- |

Q8 In driverless cars, collisions will be less common than in cars driven by humans. And driverless cars will be programmed to avoid collisions if possible. However, sometimes a collision may be unavoidable. In that situation, there will be no time for humans to take control of the car. The car will need to be programmed in advance how to respond. 
When programming driverless cars, the first important point to consider is how many people are in the car (occupants) and how many pedestrians are involved in the collision. On the next pages, you will find three examples.

| Page Break |  |
| --- | --- |

Q9
**Case 1: The same Numbers of Occupants and Pedestrians**
   In this type of collision, driverless cars need to choose either to spare Occupants or Pedestrians. Both options affect the**same numbers**of people.
  　　　　　　　　　　　　　　　　　　　　　　　　　　　　　　　　　　　　　　　(edited image from the Moral Machine study [14])

| Page Break |  |
| --- | --- |

Q10
**Case 2: More Pedestrians than Occupants**


In this type of collision, driverless cars need to choose either to spare Occupants or Pedestrians. But there are more **Pedestrians than Occupants**  

(edited image from the Moral Machine study [14])

| Page Break |  |
| --- | --- |

Q11
**Case 3: More Occupants than Pedestrians**

 In this type of collision, driverless cars need to choose either to spare Occupants or Pedestrians. But there are more **Occupants than Pedestrians.**
  
(edited image from the Moral Machine study [14])
 
 

| Page Break |  |
| --- | --- |

Q12
Now, we will introduce three possible programs for driverless cars and explain how each program responds to each of these collision scenearios.

| Page Break |  |
| --- | --- |

Q13
Program 1: **Save the Pedestrians**
**The Save the Pedestrians**program will always prioritize the safety of Pedestrians in any scenario.

(edited image from the Moral Machine study [14])

| Page Break |  |
| --- | --- |

Q14
Program 2: **Save Occupants**
  
**The Save Occupants**program will always prioritize the safety of **Occupants**in any scenario.
  
(edited image from the Moral Machine study [14])

| Page Break |  |
| --- | --- |

Q15
Program 3: **Save the most**


**The Save the most** program will save the greatest number of people in any scenario. When the same numbers of Occupants and Pedestrians are at risk, it randomizes its behavior.

(edited image from the Moral Machine study [14])

| Page Break |  |
| --- | --- |

End of Block: Welcome to Study

Start of Block: Buying A Car

Q16 Imagine now that you are planning to buy a driverless car. Imagine that the driverless car costs the same and has the same features as a regular car. However, you are given a choice in how it will be programmed to respond in the event of a collision between the car and pedestrians.

Q17 In a case of a pending collision that will involve pedestrians, how would you want your driverless car to be programmed to respond?

- **Save pedestrians**. The car will be programmed to save pedestrians, even if that means that occupants of the car will die (1)
- **Save occupants**. The car will be programmed to save occupants of the car, even if that means that pedestrians will die (2)
- **Save the most**. The car will be programmed to save the greatest number of lives, whether those are pedestrians or occupants of the car (3)
- **Random choice**. The car will be programmed to randomly choose to save either the occupants of the car or pedestrians (4)

| Page Break |  |
| --- | --- |

Q18 Imagine now that you are planning to buy a driverless car. Imagine that the driverless car costs the same and has the same features as a regular car. However, you are given a choice in how it will be programmed to respond in the event of a collision between the car and pedestrians. And imagine that **you have a young family who will often be passengers in the car.**

Q19 In a case of a pending collision that will involve pedestrians, how would you want your driverless car to be programmed to respond?

- **Save pedestrians**. The car will be programmed to save pedestrians, even if that means that occupants of the car will die (1)
- **Save occupants**. The car will be programmed to save occupants of the car, even if that means that pedestrians will die (2)
- **Save the most**. The car will be programmed to save the greatest number of lives, whether those are pedestrians or occupants of the car (3)
- **Random choice**. The car will be programmed to randomly choose to save either the occupants of the car or pedestrians (4)

| Page Break |  |
| --- | --- |

Q20 Imagine now that you are planning to buy a driverless car. Imagine that the driverless car costs the same and has the same features as a regular car. However, you are given a choice in how it will be programmed to respond in the event of a collision between the car and pedestrians. And imagine that **you have a young family who is walking a lot; they will be pedestrians on most of the days.**

Q21 In a case of a pending collision that will involve pedestrians, how would you want your driverless car to be programmed to respond?

- **Save pedestrians**. The car will be programmed to save pedestrians, even if that means that occupants of the car will die (4)
- **Save occupants**. The car will be programmed to save occupants of the car, even if that means that pedestrians will die (5)
- **Save the most**. The car will be programmed to save the greatest number of lives, whether those are pedestrians or occupants of the car (6)
- **Random choice**. The car will be programmed to randomly choose to save either the occupants of the car or pedestrians (7)

End of Block: Buying A Car

Start of Block: Utilitarian

Q22 Imagine that in ten years’ time, driverless cars are the same cost and have the same features as a regular car but are much safer than a regular car. Now, imagine that all manufacturers have programmed the car to respond to unavoidable collisions with “**Save the most**”: The car will be programmed to save the most number of lives, whether those are pedestrians or occupants of the car. No other programming option is available. How likely would you buy a driverless car programmed to **"Save the most"**if you needed to replace your existing car?

- Not at all likely (1)
- 2 (2)
- 3 (3)
- Neither likely nor unlikely (4)
- 5 (5)
- 6 (6)
- Extremely likely (7)

End of Block: Utilitarian

Start of Block: Save pedestrians

Q23 Imagine that in ten years’ time, driverless cars are the same cost and have the same features as a regular car but are much safer than a regular car. Now, imagine that all manufacturers have programmed the car to respond to unavoidable collisions with “Save the pedestrians”: The car will be programmed to save pedestrians, even if that means that occupants of the car will die. No other programming option is available. How likely would you buy a driverless car programmed to "Save the pedestrians" if you needed to replace your existing car?

- Not at all likely (1)
- 2 (2)
- 3 (3)
- Neither likely nor unlikely (4)
- 5 (5)
- 6 (6)
- Extremely likely (7)

End of Block: Save pedestrians

Start of Block: Save occupants

Q23 Imagine that in ten years’ time, driverless cars are the same cost and have the same features as a regular car but are much safer than a regular car. Now, imagine that all manufacturers have programmed the car to respond to unavoidable collisions with “Save the occupants”: The car will be programmed to save occupants of the car, even if that means that pedestrians will die. No other programming option is available. How likely would you buy a driverless car programmed to "Save the occupants" if you needed to replace your existing car?

- Not at all likely (1)
- 2 (2)
- 3 (3)
- Neither likely nor unlikely (4)
- 5 (5)
- 6 (6)
- Extremely likely (7)

End of Block: Save occupants

Start of Block: Quantity of priority ( Utilitarian less than 4 and Save Occupants more than 4

Q28 You have indicated that you would want a driverless car to prioritise the safety of occupants.
 Please indicate on the scale below how much priority you would want the car to give to occupants.

Q170 When there is only me in the car, I want my car to spare me unless there are more than...

- 2 pedestrians (75)
- 3 pedestrians (76)
- 4 pedestrians (77)
- 5 pedestrians (78)
- 6 pedestrians (79)
- 7 pedestrians (80)
- Always save me (82)

| Page Break |  |
| --- | --- |

Q171 When there is a me and a family/friend in the car, I want my car to spare us unless there are more than...

- 3 pedestrians (49)
- 4 pedestrians (50)
- 5 pedestrians (51)
- 6 pedestrians (52)
- 7 pedestrians (53)
- 8 pedestrians (54)
- Always save us (56)

End of Block: Quantity of priority ( Utilitarian less than 4 and Save Occupants more than 4

Start of Block: moral Intution

Q24 Imagine that in the future, the government has introduced a policy for how driverless cars should respond in the event of an inevitable collision. **If all driverless cars were going to be programmed the same way**, how should they be programmed from your perspective? Remember that you could be either an occupant of a car or a pedestrian.

- **Save pedestrians**: The car will be programmed to save pedestrians, even if that means that occupants of the car will die (1)
- **Save occupants**: The car will be programmed to save occupants of the car, even if that means that pedestrians will die (2)
- **Save the most**: The car will be programmed to save the greatest number of lives, whether those are pedestrians or occupants of the car (3)
- **Random choice**: The car will be programmed to randomly choose to save either the occupants of the car or pedestrians (4)

End of Block: moral Intution

Start of Block: Price: sample

Q25 Finally, we want you to choose between a series of different cars. We will present you with two cars and we want you to indicate which one you would choose. The cars will differ in how much they cost and what they are programmed. The three options for programming are the ones you are already familiar with: save the pedestrians, save the most, and save the occupants. But you can see them below explained again.    ･**Save pedestrians**: The car will be programmed to save pedestrians, even if that means that occupants of the car will die   ･**Save occupants**: The car will be programmed to save occupants of the car, even if that means that pedestrians will die   ･**Save the most**: The car will be programmed to save the most number of lives, whether those are pedestrians or occupants of the car

| Page Break |  |
| --- | --- |

Q26 On each trial, you will be presented with two cars just like pictured below. The prize of the care is indicated by the price tag, the programming of the car is indicated by the computer symbol. 
Then you are asked which car you would prefer buying, using the scale provided underneath (there are 36 questions).

Q27


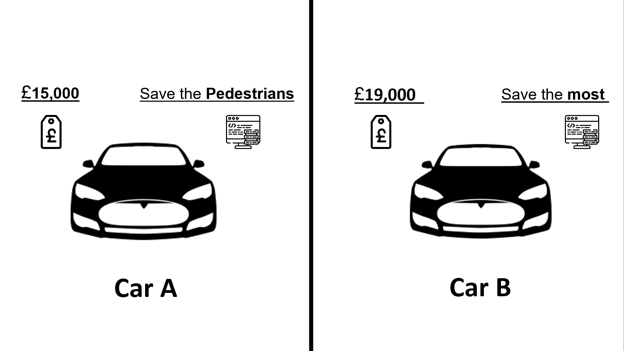


Q28 To make sure that you understand the pictures, please indicate how much Car A costs?

- £15,000 (1)
- £19,000 (2)
- £23,000 (3)

Q29 Please indicate which programming Car B has?

- Save the pedestrians (1)
- Save the most (2)
- Save the occupants (3)

End of Block: Price: sample

Start of Block: wrong choice

Q30 You made a wrong choice. Please answer the question again.

End of Block: wrong choice

Start of Block: price Sample 2

Q31 On each trial, you will be presented with two cars just like pictured below. The price of the care is indicated by the price tag, the programming of the car is indicated by the computer symbol. 
Then you are asked which car you would prefer buying, using the scale provided underneath. 


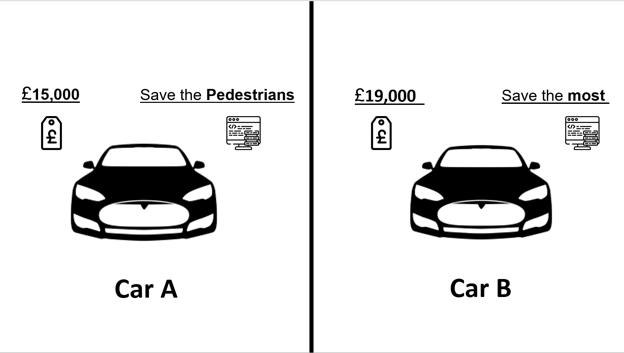


Q33 To make sure that you understand the pictures, please indicate how much Car A costs?

- £15,000 (1)
- £19,000 (2)
- £23,000 (3)

Q34 Please indicate which programming Car B has?

- Save the pedestrians (1)
- Save the most (2)
- Save the occupants (3)

End of Block: price Sample 2

Start of Block: Car Choice 1

Q35
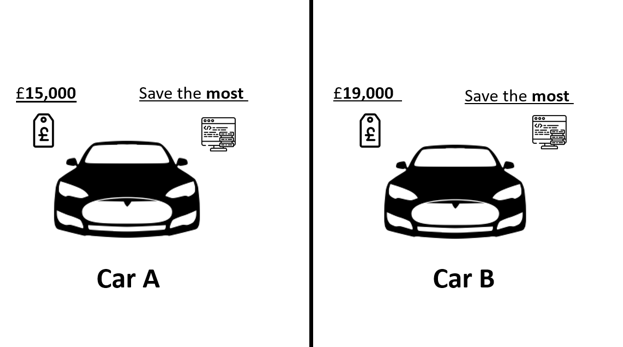

 Which car would you buy?

- A (1)
- B (2)

End of Block: Car Choice 1

Start of Block: 2

Q36
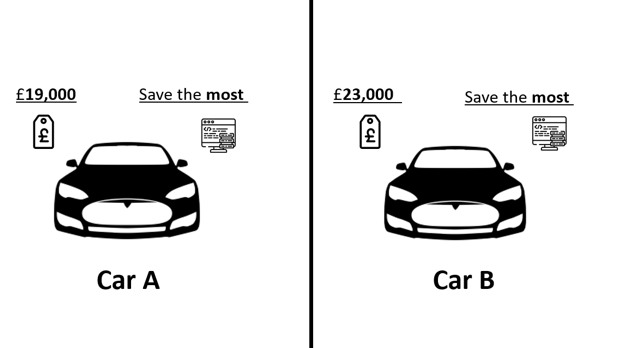
 Which car would you buy?

- A (1)
- B (2)

End of Block: 2

Start of Block: 3

Q37
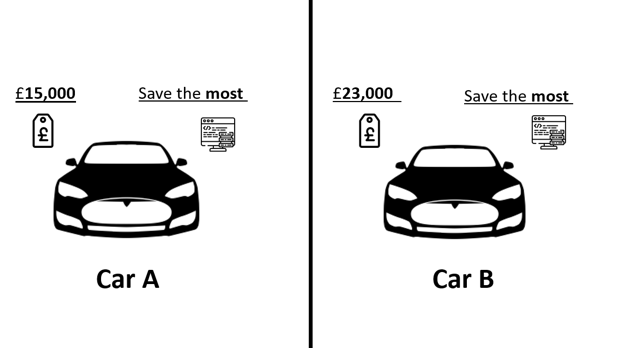
 Which car would you buy?

- A (1)
- B (2)

End of Block: 3

Start of Block: 4

Q38
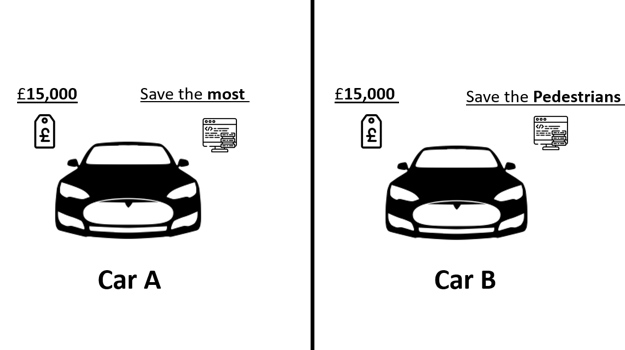
 Which car would you buy?

- A (1)
- B (2)

End of Block: 4

Start of Block: 5

Q39
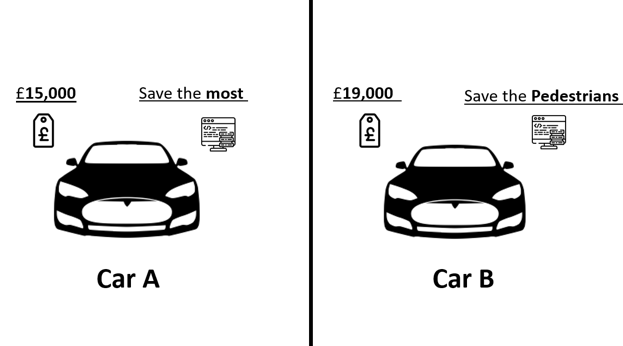
 Which car would you buy?

- A (1)
- B (2)

End of Block: 5

Start of Block: 6

Q40
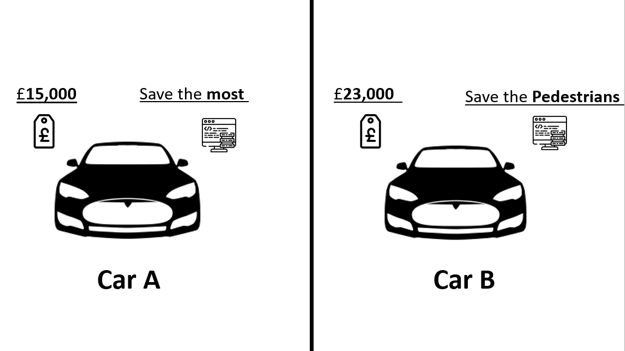
 Which car would you buy?

- A (1)
- B (2)

End of Block: 6

Start of Block: 7

Q41
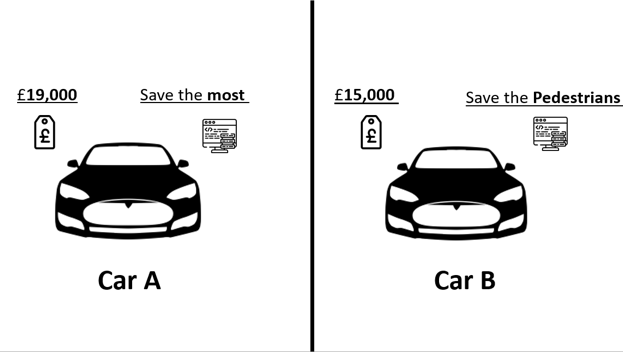
 Which car would you buy?

- A (1)
- B (2)

End of Block: 7

Start of Block: 8

Q42
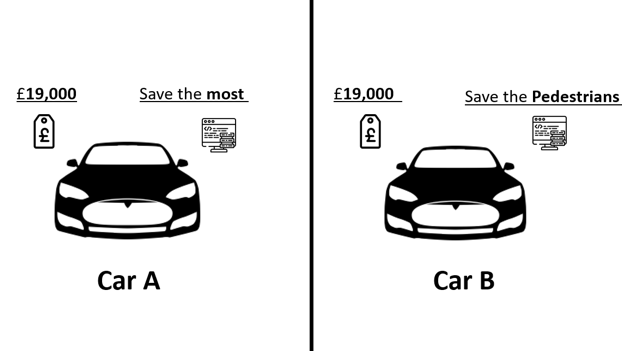
 Which car would you buy?

- A (1)
- B (2)

End of Block: 8

Start of Block: 9

Q43
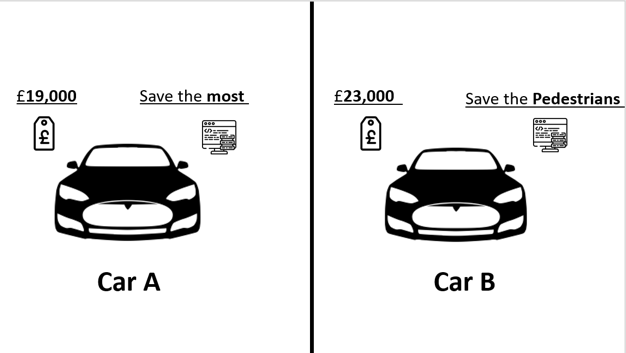
 Which car would you buy?

- A (1)
- B (2)

End of Block: 9

Start of Block: 10

Q44
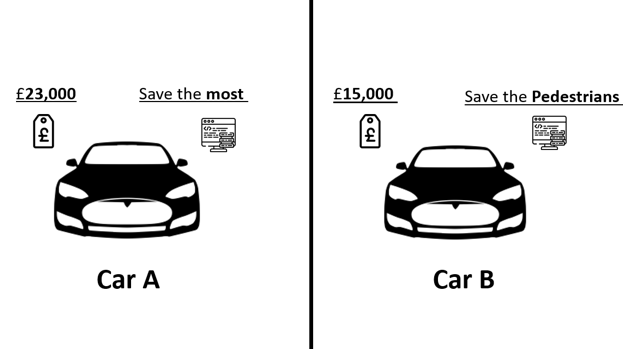
 Which car would you buy?

- A (1)
- B (2)

End of Block: 10

Start of Block: 11

Q45
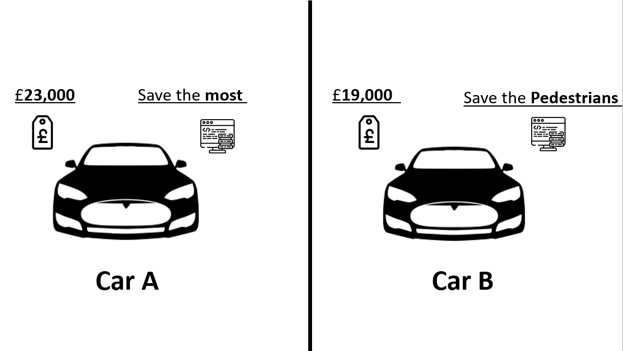
 Which car would you buy?

- A (1)
- B (2)

End of Block: 11

Start of Block: 12

Q46
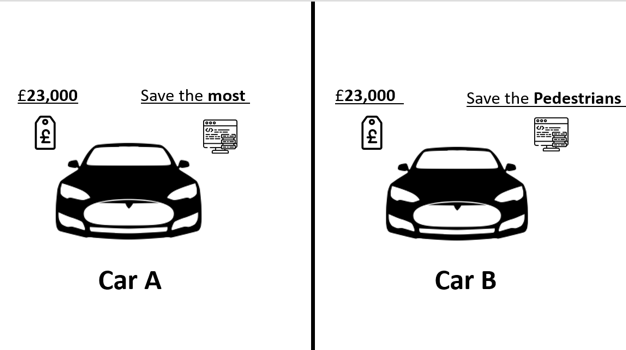
 Which car would you buy?

- A (1)
- B (2)

End of Block: 12

Start of Block: 13

Q47
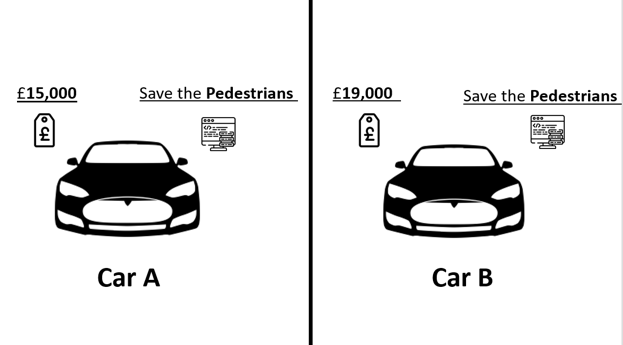
 Which car would you buy?

- A (1)
- B (2)

End of Block: 13

Start of Block: 14

Q48
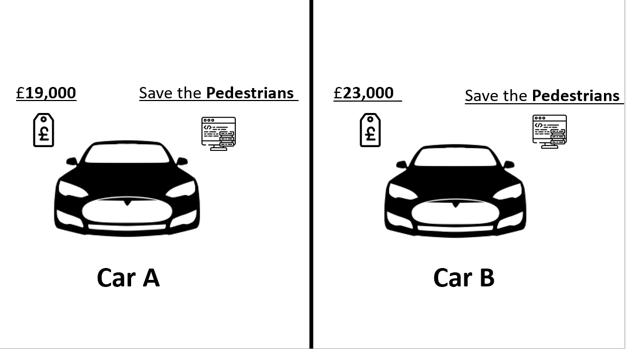
 Which car would you buy?

- A (1)
- B (2)

End of Block: 14

Start of Block: 15

Q49
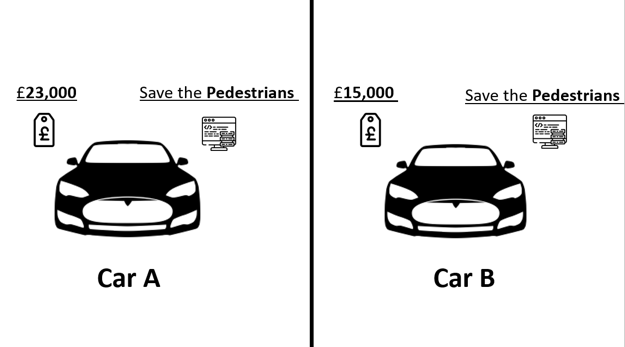
 Which car would you buy?

- A (1)
- B (2)

End of Block: 15

Start of Block: 16

Q50
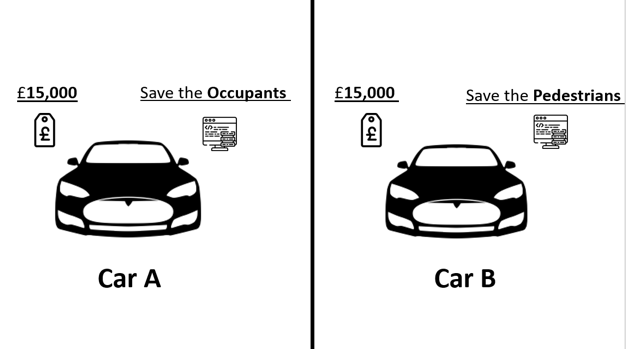
 Which car would you buy?

- A (1)
- B (2)

End of Block: 16

Start of Block: 17

Q51
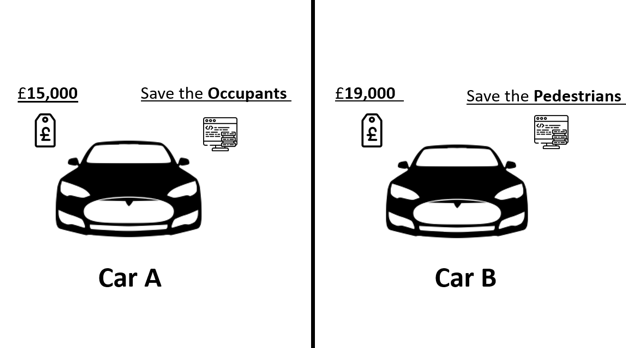
 Which car would you buy?

- A (1)
- B (2)

End of Block: 17

Start of Block: 18

Q52
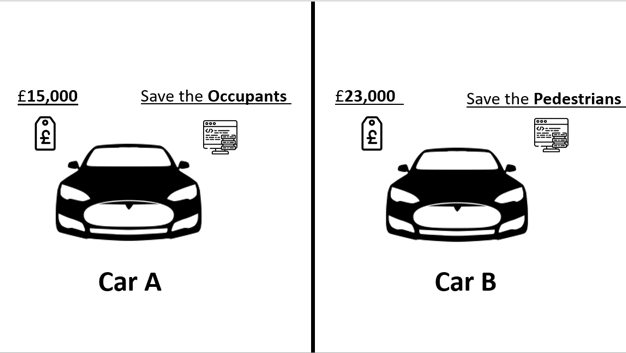
 Which car would you buy?

- A (1)
- B (2)

End of Block: 18

Start of Block: 19

Q53
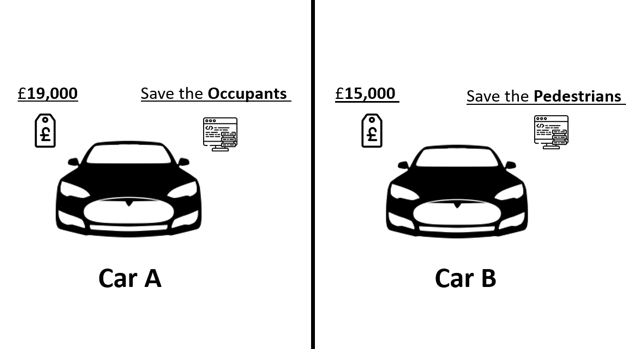
 Which car would you buy?

- A (1)
- B (2)

End of Block: 19

Start of Block: 20

Q54
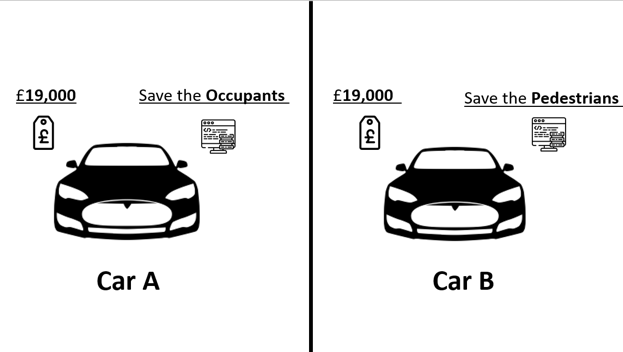
 Which car would you buy?

- A (1)
- B (2)

End of Block: 20

Start of Block: 21

Q55
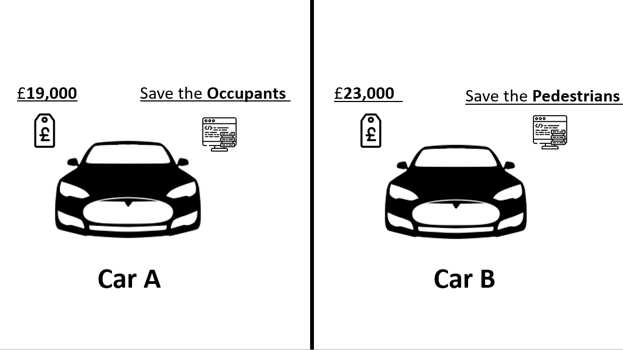
 Which car would you buy?

- A (1)
- B (2)

End of Block: 21

Start of Block: 22

Q56
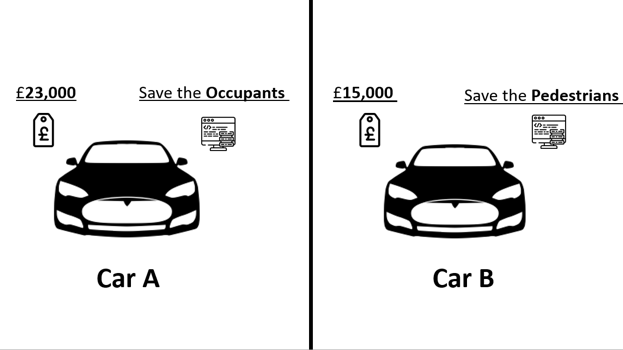
 Which car would you buy?

- A (1)
- B (2)

End of Block: 22

Start of Block: 23

Q57
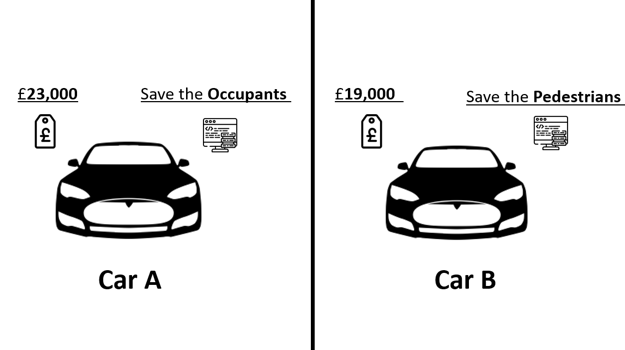
 Which car would you buy?

- A (1)
- B (2)

End of Block: 23

Start of Block: 24

Q58
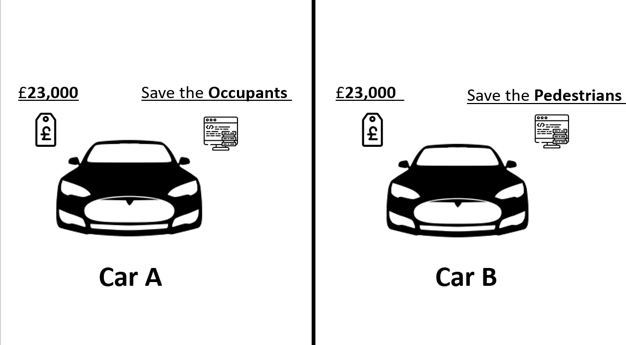
 Which car would you buy?

- A (1)
- B (2)

End of Block: 24

Start of Block: 25

Q59
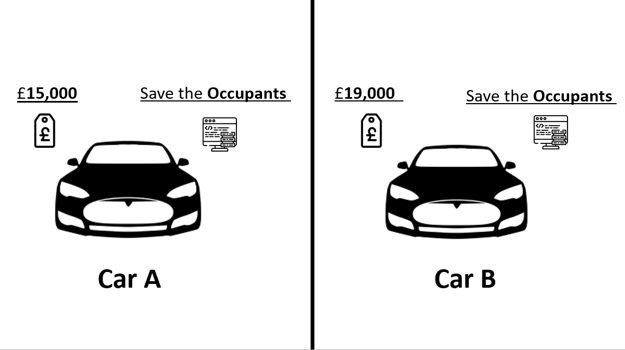
 Which car would you buy?

- A (1)
- B (2)

End of Block: 25

Start of Block: 26

Q60
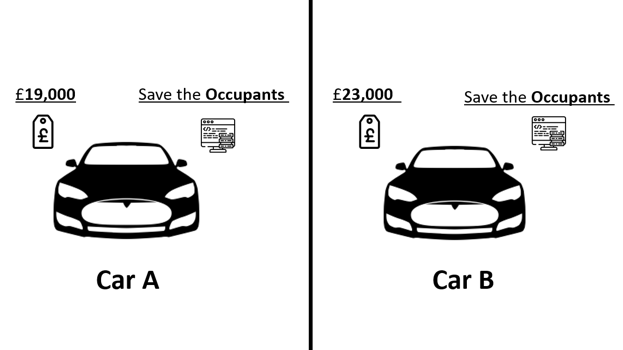
 Which car would you buy?

- A (1)
- B (2)

End of Block: 26

Start of Block: 27

Q61
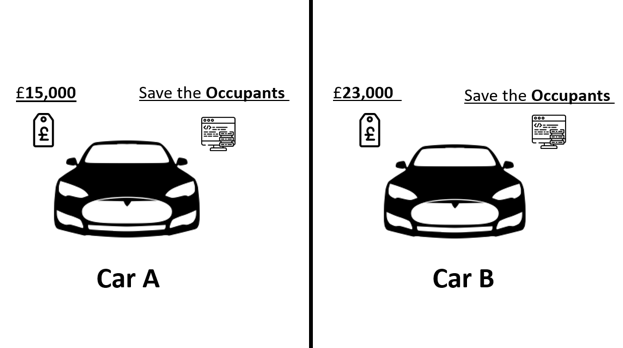
 Which car would you buy?

- A (1)
- B (2)

End of Block: 27

Start of Block: 28

Q62
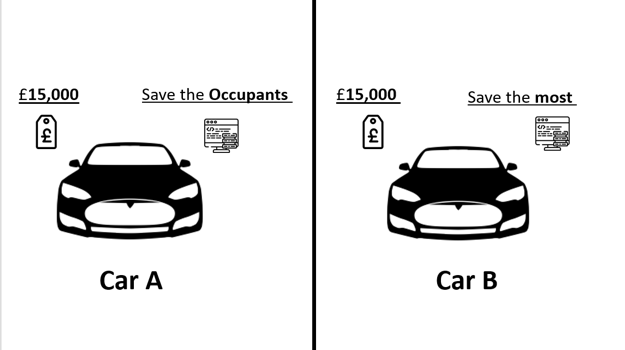
 Which car would you buy?

- A (1)
- B (2)

End of Block: 28

Start of Block: 29

Q63
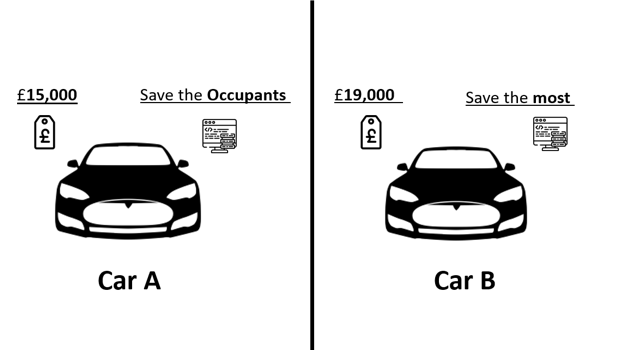
 Which car would you buy?

- A (1)
- B (2)

End of Block: 29

Start of Block: 30

Q64
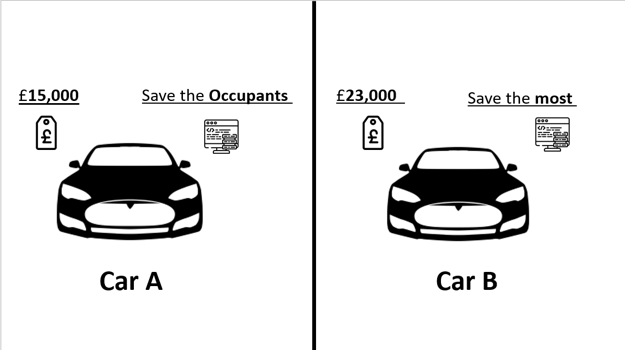
 Which car would you buy?

- A (1)
- B (2)

End of Block: 30

Start of Block: 31

Q65
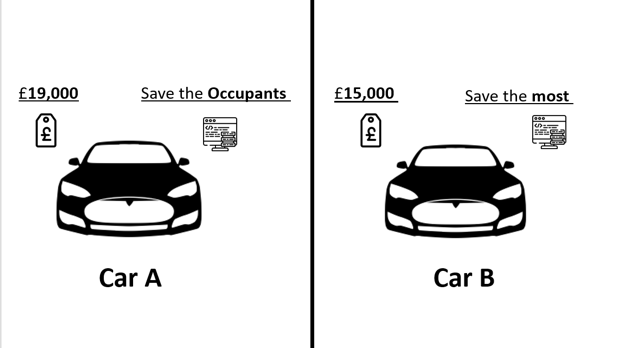
 Which car would you buy?

- A (1)
- B (2)

End of Block: 31

Start of Block: 32

Q66
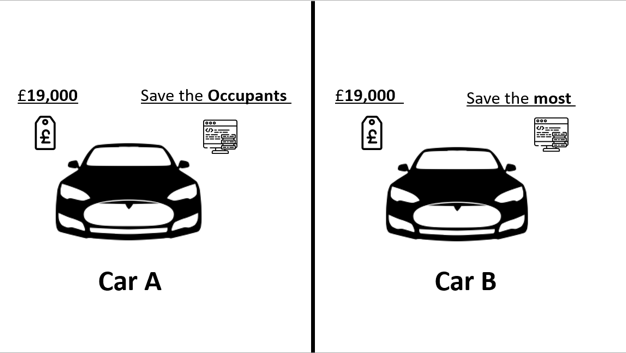
 Which car would you buy?

- A (1)
- B (2)

End of Block: 32

Start of Block: 33

Q67
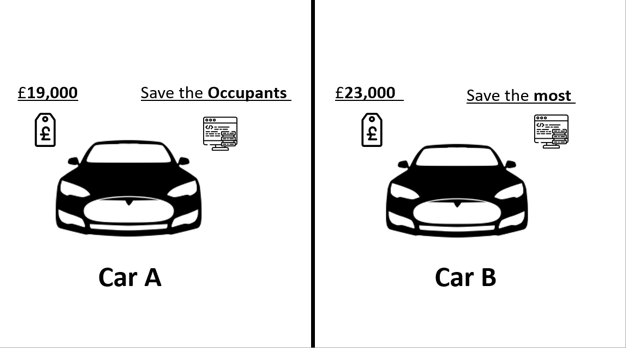
 Which car would you buy?

- A (1)
- B (2)

End of Block: 33

Start of Block: 34

Q68
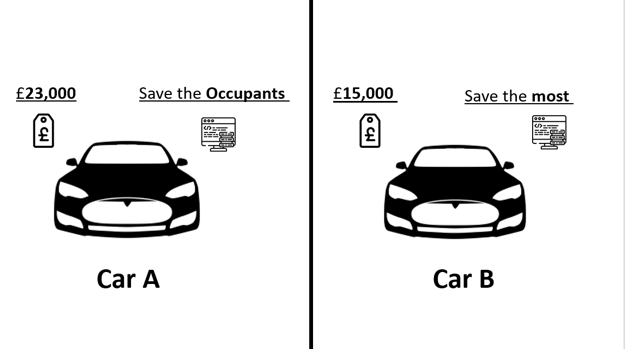
 Which car would you buy?

- A (1)
- B (2)

End of Block: 34

Start of Block: 35

Q69
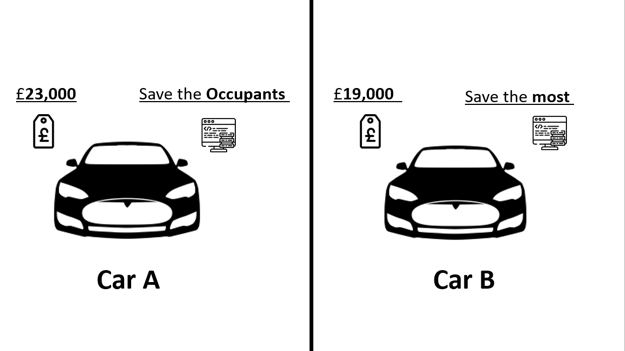
 Which car would you buy?

- A (1)
- B (2)

End of Block: 35

Start of Block: 36

Q70
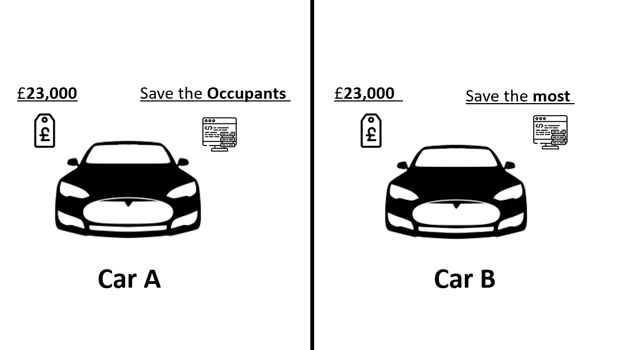
 Which car would you buy?

- A (1)
- B (2)

End of Block: 36

Start of Block: How utilitarian they are?

Q71 At the end of this study, we want to know a bit more about your moral inclinations.

| Page Break |  |
| --- | --- |

Q72 Q. If the only way to save another person’s life during an emergency is to sacrifice one’s own leg, then one is morally required to make this sacrifice.

- Strongly disagree (1)
- Disagree (2)
- Somewhat disagree (3)
- Neither agree nor disagree (4)
- Somewhat agree (5)
- Agree (6)
- Strongly agree (7)

Q73 Q. From a moral point of view, we should feel obliged to give one of our kidneys to a person with kidney failure since we do not need two kidneys to survive, but really only one to be healthy.

- Strongly disagree (1)
- Disagree (2)
- Somewhat disagree (3)
- Neither agree nor disagree (4)
- Somewhat agree (5)
- Agree (6)
- Strongly agree (7)

Q74 Q. From a moral perspective, people should care about the well-being of all human beings on the planet equally; they should not favor the well-being of people who are especially close to them either physically or emotionally.

- Strongly disagree (1)
- Disagree (2)
- Somewhat disagree (3)
- Neither agree nor disagree (4)
- Somewhat agree (5)
- Agree (6)
- Strongly agree (7)

Q75 Q. It is just as wrong to fail to help someone as it is to actively harm them yourself.

- Strongly disagree (1)
- Disagree (2)
- Somewhat disagree (3)
- Neither agree nor disagree (4)
- Somewhat agree (5)
- Agree (6)
- Strongly agree (7)

Q76 Q. It is morally wrong to keep money that one doesn’t really need if one can donate it to causes that provide effective help to those who will benefit a great deal.

- Strongly disagree (1)
- Disagree (2)
- Somewhat disagree (3)
- Neither agree nor disagree (4)
- Somewhat agree (5)
- Agree (6)
- Strongly agree (7)

Q77 Q. It is morally right to harm an innocent person if harming them is a necessary means to helping several other innocent people.

- Strongly disagree (1)
- Disagree (2)
- Somewhat disagree (3)
- Neither agree nor disagree (4)
- Somewhat agree (5)
- Agree (6)
- Strongly agree (7)

Q78 Q. If the only way to ensure the overall well-being and happiness of the people is through the use of political oppression for a short, limited period, then political oppression should be used.

- Strongly disagree (1)
- Disagree (2)
- Somewhat disagree (3)
- Neither agree nor disagree (4)
- Somewhat agree (5)
- Agree (6)
- Strongly agree (7)

Q79 Q. It is permissible to torture an innocent person if this would be necessary to provide information to prevent a bomb going off that would kill hundreds of people.

- Strongly disagree (1)
- Disagree (2)
- Somewhat disagree (3)
- Neither agree nor disagree (4)
- Somewhat agree (5)
- Agree (6)
- Strongly agree (7)

Q80 Q. Sometimes it is morally necessary for innocent people to die as collateral damage—if more people are saved overall.

- Strongly disagree (1)
- Disagree (2)
- Somewhat disagree (3)
- Neither agree nor disagree (4)
- Somewhat agree (5)
- Agree (6)
- Strongly agree (7)

End of Block: How utilitarian they are?

Start of Block: Thank You

Q189 Thank you for participating. Your secret completion code is:   **59110E0C**  

End of Block: Thank You

Start of Block: Give up

Q131 We will skip this question, no worries!

End of Block: Give up

**Material 2: Japanese questionnaire**

Start of Block: Informed Consent

Q0  参加者の皆様へのお願い     **【調査の目的について】**
 本調査は皆様の自動運転車に対する考え方、および購買意欲を把握することを目的としています。 以下の注意事項を読み、本調査の趣旨を理解いただいたうえで、調査にご参加いただければ幸いです。
 
**【調査の内容について】**
 アンケートの回答には約15分かかる予定です。 事前知識は必要ございません。一例として、将来、自動運転車が避けられない事故に直面した際、どのような設定を組み入れるべきか、また組み入れてほしいか、といった質問をお伺いします。 アンケートへの参加は皆様の自由であり、参加しなくても、参加を途中で中止しても、それによって皆様に不都合が生じることはありません。しかし、アンケートを途中で中止した場合、謝礼はお支払いされません。 なお、アンケートには質問文を適切にお読みいただいているかを確認する「チェック質問」がございます。正しく回答いただけなかった場合、アンケートは強制終了されます。またその場合、謝礼はお支払いされません。 

 **【皆様より得られたデータについて】**
 すべての回答は匿名化され、個人が特定されない形に加工して、学会や学術誌、HP等で公表いたします。 皆様から得られたデータは、調査終了後、最低三年間は保存されます。調査の参加に同意いただくことでデータの取り扱いにも同意したことになります。その点をご了承いただきますよう、よろしくお願い申し上げます。 
 
**【研究組織（調査主体）について】**
 本調査はオックスフォード大学の倫理審査委員会の承認を得て実施しております。調査代表者はドミニク・ウィルキンソン（所属：オックスフォード大学上廣応用倫理研究センター・教授）です。質問等は以下の連絡先までご連絡ください。

 連絡先：ktakaguchi4986@gmail.com
 

 本調査は日本在住の18歳以上の方を対象としております。上記の点に同意いただける場合は以下のチェックボックスをクリックしてアンケートへお進みください。
 
（調査の最後にパスコードを表示いたします。そのコードを正しくCrowdworksの二つ目の質問にご入力いただかなければ、報酬は支払われません。）

- 私は18歳以上であり、アンケートの参加に同意します (2)

End of Block: Informed Consent

Start of Block: Block 2

Q１ Crowdworksのアカウント名を記入してください

________________________________________________________________

End of Block: Block 2

Start of Block: Attention Check

Q2 回答する際は、質問の条件や内容をよく読んでいただく必要があります。過去の調査によると、参加者の中には質問文を読まない方がいることがわかっています。以下の2つの質問は、回答者が実際に質問文を読んでいるかを確認するための質問です。あなたがもしこの質問文を読んでいれば、1問目の質問では6を、2問目の質問ではその数字を2にで割った数字を回答してください。正しく回答していただいた方のみが次に進むことができます。

Q3 私はたとえ物価が高くても、様々な文化的体験ができる街に住みたい。

- 強く賛成 (1)
- 1 (2)
- 2 (3)
- 3 (4)
- 4 (5)
- 5 (6)
- 6 (7)

Q4 私は小さい街より大きい街に住みたい。

- 強く賛成 (1)
- 1 (2)
- 2 (3)
- 3 (4)
- 4 (5)
- 5 (6)
- 6 (7)

End of Block: Attention Check

Start of Block: Sorry

Q5 先ほどの質問で正しい回答をいただけなかったため、調査は終了させていただきます。ご協力ありがとうございました。

End of Block: Sorry

Start of Block: Welcome to Study

Q6
 
**アンケートにご参加いただきありがとうございます。**
  
このアンケートでは、皆様の自動運転車に対する考え方、および購買意欲について、以下の前提条件をもとに、あなたのお考えをお伺いします。

 【前提条件】
 将来的に、自動運転車は一般的な車（人が運転する車）より安全になり、90－94％の交通事故を未然に防ぐことができると予想されています。 あなたは目的地に到着するまで、一切運転をする必要がありません。しかし、衝突が避けられない場合もご自身で運転することができません。皆様がどのような設定の車を欲しいか質問していきます。

 このアンケートでは、自動運転システムは誤操作を起こさずに安全に作動し、ハッキングされるリスクもないと仮定してください。また自動運転車を所有したとしても、個人情報が漏洩したり、事故が起こった際に責任を問われたりすることもないと仮定してください。

| Page Break |  |
| --- | --- |

Q7 今から10年後、一般車と価格が同じで、見た目も同じであるにもかかわらず、一般車よりずっと安全な自動運転車が発売されたとします。
 
**あなたが車の買い替えを検討している場合、自動運転車を購入したいと思いますか？**
 

- 全く思わない (8)
- 2 (2)
- 3 (3)
- どちらともいえない (4)
- 5 (5)
- 6 (6)
- 強く思う (7)

| Page Break |  |
| --- | --- |

Q8 【事故の可能性】
自動運転車は人が運転する車より安全で、交通事故を回避するよう設定（プログラム）されていますが、時に交通事故に巻き込まれることもあります。事故に直面した時、搭乗者（運転席に座っている人）が運転を代わる時間はないため、自動運転車がどのように作動するかを事前に設定しておかなければなりません。


 その設定においてまず考えなければならない大事な点は、車に何人が乗っていて（搭乗者は何人か）、事故に巻き込まれる歩行者は何人か、というものです。次に、3つの交通事故の事例について考えていただきます。

| Page Break |  |
| --- | --- |

Q9
 
**事例1）搭乗者と歩行者の人数が同じ場合**
  この交通事故の事例では、搭乗者と歩行者の数が同じで、自動運転車はどちらを助けるか選択しなければなりません。どちらを選択しても、**同じ数の人**が助かります。

  (edited image from the Moral Machine study [14])

| Page Break |  |
| --- | --- |

Q10
 
**事例２）搭乗者よりも歩行者の方が多い場合**
 
この交通事故の事例でも、自動運転車は搭乗者と歩行者のどちらを助けるかを選択しなければなりません。しかし、**搭乗者よりも歩行者の方が多いという**状況です。
  
 (edited image from the Moral Machine study [14])


  

| Page Break |  |
| --- | --- |

Q11
**事例３）歩行者よりも搭乗者の方が多い場合**
 
この交通事故の事例でも、自動運転車は搭乗者と歩行者のどちらを助けるかを選択しなければなりません。しかし、**歩行者よりも搭乗者の方が多いという**状況です。
 
  (edited image from the Moral Machine study [14])
  
 
 

| Page Break |  |
| --- | --- |

Q12 次に、これらの交通事故において考えられる自動運転車の設定を三つ紹介し、それぞれの設定が交通事故の場面でどう作動するかについて説明します。

| Page Break |  |
| --- | --- |

Q13
 
設定１）**歩行者の救命**
 
  **歩行者の救命の設定**では、常に歩行者の安全を優先します。

(edited image from the Moral Machine study [14])

| Page Break |  |
| --- | --- |

Q14
設定2）**搭乗者の救命**
 
**搭乗車の救命**の設定では、常に搭乗者の安全を優先します。

(edited image from the Moral Machine study [14])

| Page Break |  |
| --- | --- |

Q15
 
 設定３）**最大多数の救命**


**最大多数の救命**の設定では、常に最も多くの人を助けることができます。もし同じ人数の搭乗者と歩行者が危険にさらされている場合、この設定はランダムに作動します。

   (edited image from the Moral Machine study [14])

| Page Break |  |
| --- | --- |

End of Block: Welcome to Study

Start of Block: Buying A Car

Q16 あなたは自動運転車の購入を検討していると想像してください。自動運転車は一般車と同じ価格、同じような見た目をしています。しかし、車と歩行者が衝突する場面で、その車がどう作動するかをあなたが設定できます。

Q17 歩行者を巻き込む衝突事故が起こった場合、あなたは自分の自動運転車がどう作動するよう設定したいですか？

- **［歩行者の救命］**　歩行者の救命を最優先する設定 (1)
- **［搭乗者の救命］**　搭乗者の救命を最優先する設定 (2)
- **［最大多数の救命］**　歩行者と搭乗者の人数を比較し、最も多くの人を救命する設定 (3)
- **［無作為な救命］**　歩行者と搭乗者のどちらを助けるかを無作為に決定する設定 (4)

| Page Break |  |
| --- | --- |

Q18 前の質問に続き、あなたは自動運転車の購入を検討していて、自動運転車は一般車と同じ価格、同じような見た目をしていると想像してください。しかし、車と歩行者が衝突する場面で、その車がどう作動するかをあなたが設定できます。ここでは、**あなたには子供がいて、車にその子供がよく同乗すると想像してください。**

Q19 歩行者を巻き込む衝突事故が起こった場合、あなたは自分の自動運転車がどう作動するよう設定したいですか？

- **［歩行者の救命］**　歩行者の救命を最優先する設定 (1)
- **［搭乗者の救命］**　搭乗者の救命を最優先する設定 (2)
- **［最大多数の救命］**　歩行者と搭乗者の人数を比較し、最も多くの人を救命する設定 (3)
- **［無作為な救命］**　歩行者と搭乗者のどちらを助けるかを無作為に決定する設定 (4)

| Page Break |  |
| --- | --- |

Q20 前の質問に続き、あなたは自動運転車の購入を検討していて、自動運転車は一般車と同じ価格、同じような見た目をしていると想像してください。しかし、車と歩行者が衝突する場面で、その車がどう作動するかをあなたが設定できます。ここでは、**あなたにはよく散歩する子供がいて、その子供はほとんど毎日、歩行者として近くの道路を利用すると想像してください。**

Q21 歩行者を巻き込む衝突事故が起こった場合、あなたは自分の自動運転車がどう作動するよう設定したいですか？

- **［歩行者の救命］**　歩行者の救命を最優先する設定 (4)
- **［搭乗者の救命］**　搭乗者の救命を最優先する設定 (5)
- **［最大多数の救命］**　歩行者と搭乗者の人数を比較し、最も多くの人を救命する設定 (6)
- **［無作為な救命］**　歩行者と搭乗者のどちらを助けるかを無作為に決定する設定 (7)

End of Block: Buying A Car

Start of Block: Utilitarian

Q22 今から10年後、一般車と価格が同じで、見た目も同じであるにもかかわらず、一般車よりずっと安全な自動運転車が発売されるとします。あなたは車の買い替えを考えていますが、すべての自動運転車には**［最大多数の救命］**が設定されています。したがって、事故が避けられない場合は、歩行者と搭乗者の数を比較し、多くの人が助かるように作動します。あなたはこの**［最大多数の救命］**型の自動運転車をどの程度購入したいと思いますか？

- 全く思わない (1)
- 2 (2)
- 3 (3)
- どちらともいえない (4)
- 5 (5)
- 6 (6)
- 強く思う (7)

End of Block: Utilitarian

Start of Block: Save pedestrians

Q23 今から10年後、一般車と価格が同じで、見た目も同じであるにもかかわらず、一般車よりずっと安全な自動運転車が発売されるとします。あなたは車の買い替えを考えていますが、すべての自動運転車には**［歩行者の救命］**が設定されています。したがって、事故が避けられない場合、歩行者の救命を最優先するように作動します。あなたはこの**［歩行者の救命］**型の自動運転車をどの程度購入したいと思いますか？

- 全く思わない (1)
- 2 (2)
- 3 (3)
- どちらともいえない (4)
- 5 (5)
- 6 (6)
- 強く思う (7)

End of Block: Save pedestrians

Start of Block: Save occupants

Q23 今から10年後、一般車と価格が同じで、見た目も同じであるにもかかわらず、一般車よりずっと安全な自動運転車が発売されるとします。あなたは車の買い替えを考えていますが、すべての自動運転車には**［搭乗者の救命］**が設定されています。したがって、事故が避けられない場合、搭乗者の救命を最優先するように作動します。あなたはこの**［搭乗者の救命］**型の自動運転車をどの程度購入したいと思いますか？

- 全く思わない (1)
- 2 (2)
- 3 (3)
- どちらともいえない (4)
- 5 (5)
- 6 (6)
- 強く思う (7)

End of Block: Save occupants

Start of Block: moral Intution

Q24 将来、自動運転車が事故を避けられない状況でどう作動するかについて、政府が方針を示すと想像してください。**もし、すべての自動運転車にある特定の設定が組み込まれるとしたら**、あなたはどの設定が最適であると考えますか？あなた自身が搭乗者になることもあれば、歩行者になることもある点に留意してご回答ください。

- **［歩行者の救命］**　歩行者の救命を最優先する設定 (1)
- **［搭乗者の救命］**　搭乗者の救命を最優先する設定 (5)
- **［最大多数の救命］**　歩行者と搭乗者の人数を比較し、最も多くの人を救命する設定 (6)
- **［無作為な救命］**　歩行者と搭乗者のどちらを助けるかを無作為に決定する設定 (7)

End of Block: moral Intution

Start of Block: Price: sample

Q25 最後に、二つの種類の車を提示するので、どちらの車を購入したいと思うかお答えください。その二つの車は、異なる価格と事故に対応する設定が提示されています。設定に関しては、先ほどの質問の選択肢にもあった以下の3種類が提示されます。もう一度、それぞれの設定について説明をします。
 


 ・**［歩行者の救命］**　車の搭乗者が犠牲になったとしても、歩行者の救命を最優先する設定
 
・**［搭乗者の救命］**歩行者が犠牲になったとしても、車の搭乗者の救命を最優先する設定
 
・**［最大多数の救命］**　歩行者と搭乗者の人数を比較し、最も多くの人を救命する設定。

| Page Break |  |
| --- | --- |

Q26 以下の画像のように、それぞれの質問で二つの車が提示されます。車の左上には値段のタグのアイコンとその車の価格が、右上にはコンピューターのアイコンとその車の設定が表示されています。


それぞれの質問でどちらの車を購入したいかをお答えください（36問ございます）。

Q27


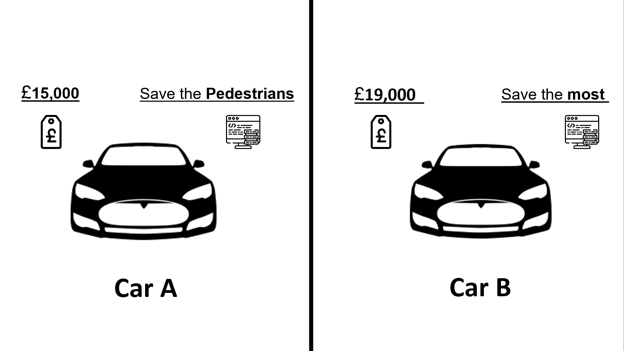


Q28 これは、画像にある車の価格と設定を正しく理解されているかを確認するための例題です。
車Aはいくらでしょうか？

- 225万円 (1)
- 285万円 (2)
- 345万円 (3)

Q29 車Bはどの設定がされているでしょうか？

- ［歩行者の救命］ (1)
- ［搭乗者の救命］ (2)
- ［最大多数の救命］ (3)

End of Block: Price: sample

Start of Block: wrong choice

Q30 回答いただいた選択肢が正しくないため、もう一度例題にお答えいただきます。

End of Block: wrong choice

Start of Block: price Sample 2

Q31 以下の画像のように、それぞれの質問で二つの車が提示されます。車の左上には値段のタグのアイコンとその車の価格が、右上にはコンピューターのアイコンとその車の設定が表示されています。
 
それぞれの質問でどちらの車を購入したいかお答えください。
  
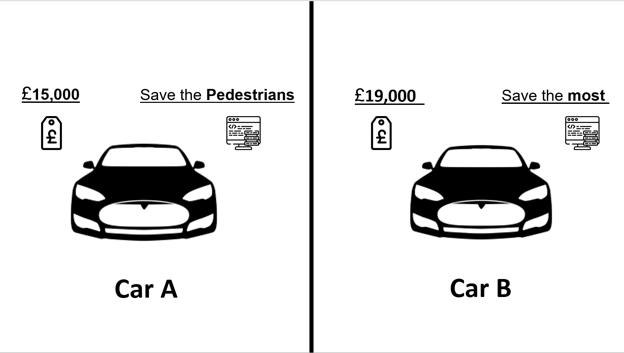


Q33 まずは画像にある車の価格と設定を正しく理解されているか、確認するための例題を出します。車Aはいくらでしょうか？

- 225万円 (1)
- 285万円 (2)
- 345万円 (3)

Q34 車Bはどの設定がされているでしょうか？

- ［歩行者の救命］ (1)
- ［搭乗者の救命］ (2)
- ［最大多数の救命］ (3)

End of Block: price Sample 2

Start of Block: Car Choice 1

Q35
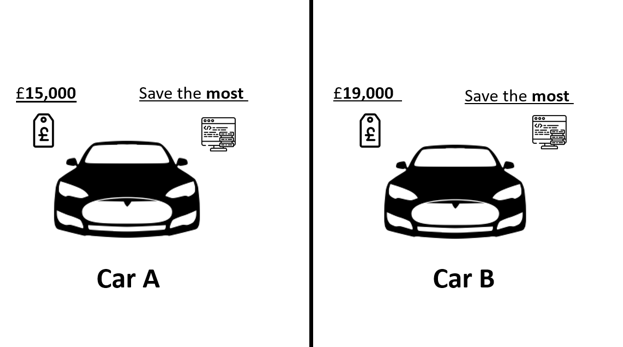
 どちらの車を購入したいですか？

- A (1)
- B (2)

End of Block: Car Choice 1

Start of Block: 2

Q36
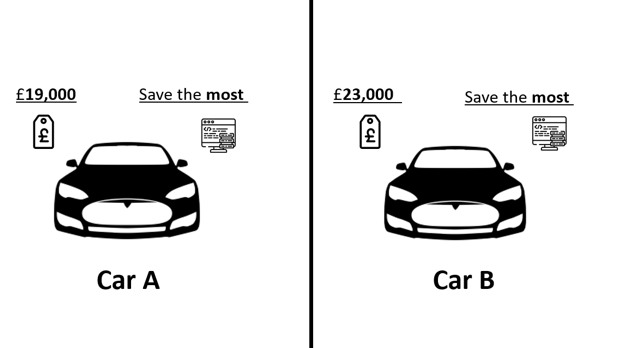
どちらの車を購入したいですか？

- A (1)
- B (2)

End of Block: 2

Start of Block: 3

Q37
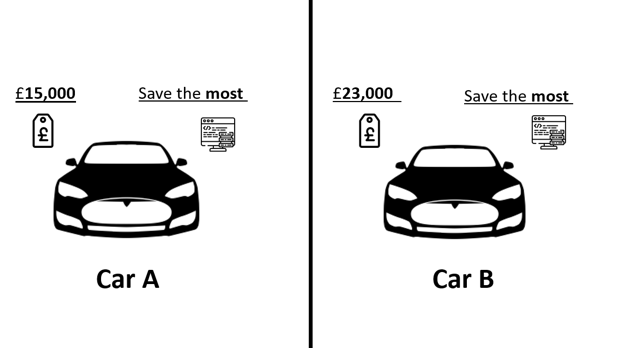
どちらの車を購入したいですか？

- A (1)
- B (2)

End of Block: 3

Start of Block: 4

Q38
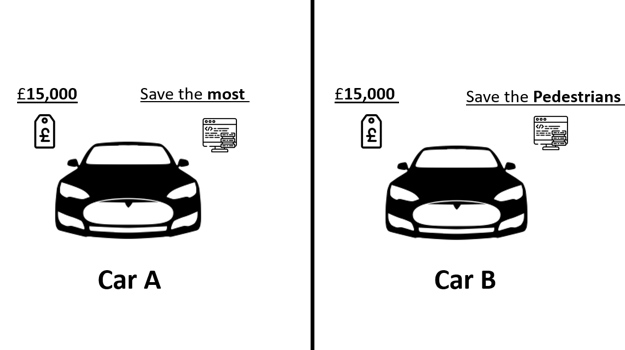
どちらの車を購入したいですか？

- A (1)
- B (2)

End of Block: 4

Start of Block: 5

Q39
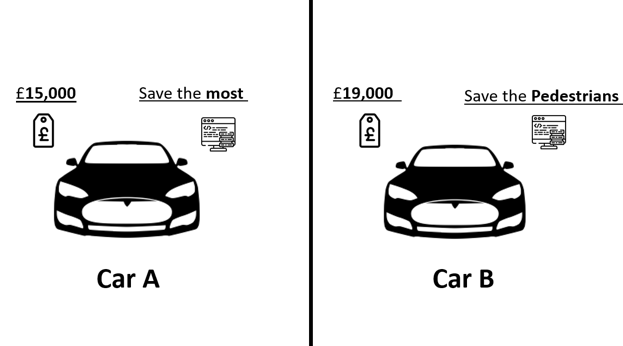
どちらの車を購入したいですか？

- A (1)
- B (2)

End of Block: 5

Start of Block: 6

Q40
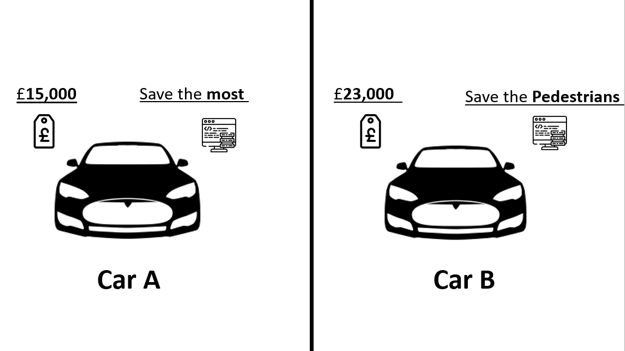
どちらの車を購入したいですか？

- A (1)
- B (2)

End of Block: 6

Start of Block: 7

Q41
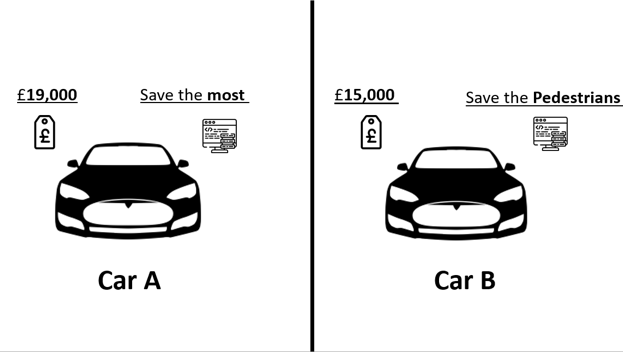
どちらの車を購入したいですか？

- A (1)
- B (2)

End of Block: 7

Start of Block: 8

Q42
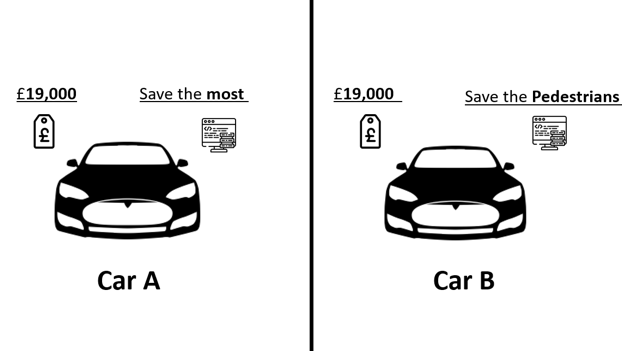
どちらの車を購入したいですか？

- A (1)
- B (2)

End of Block: 8

Start of Block: 9

Q43
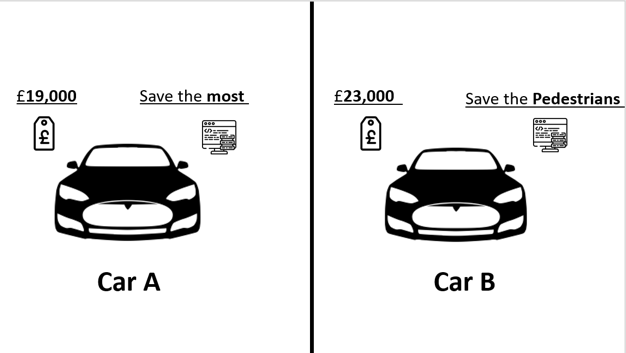
どちらの車を購入したいですか？

- A (1)
- B (2)

End of Block: 9

Start of Block: 10

Q44
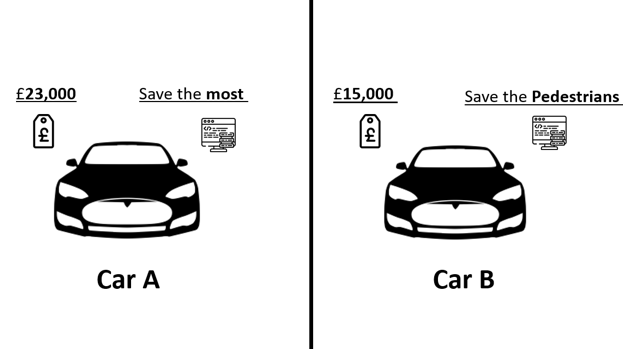
どちらの車を購入したいですか？

- A (1)
- B (2)

End of Block: 10

Start of Block: 11

Q45
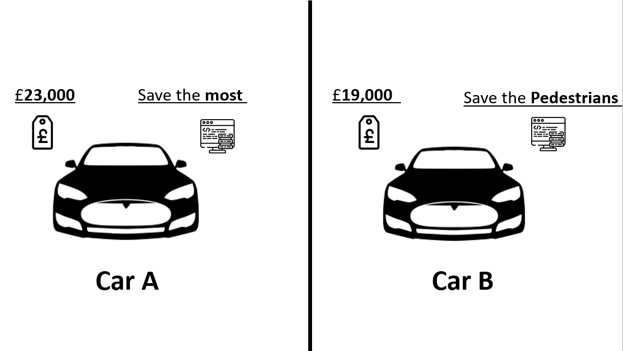
どちらの車を購入したいですか？

- A (1)
- B (2)

End of Block: 11

Start of Block: 12

Q46
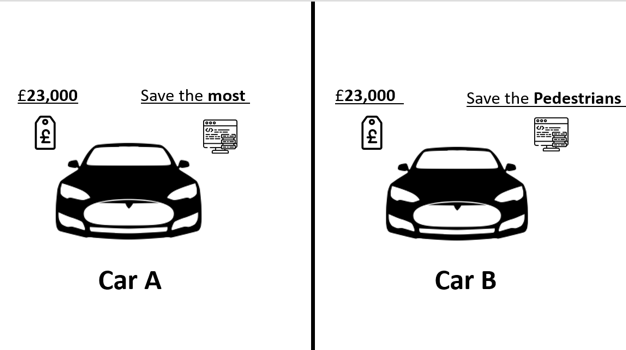
どちらの車を購入したいですか？

- A (1)
- B (2)

End of Block: 12

Start of Block: 13

Q47
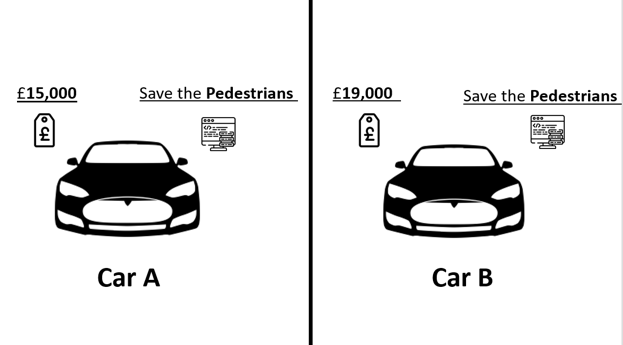
どちらの車を購入したいですか？

- A (1)
- B (2)

End of Block: 13

Start of Block: 14

Q48
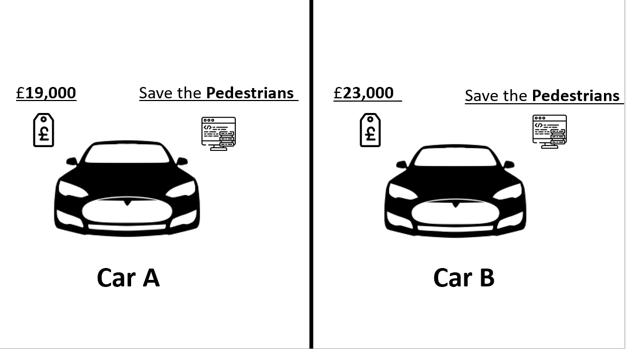
どちらの車を購入したいですか？

- A (1)
- B (2)

End of Block: 14

Start of Block: 15

Q49
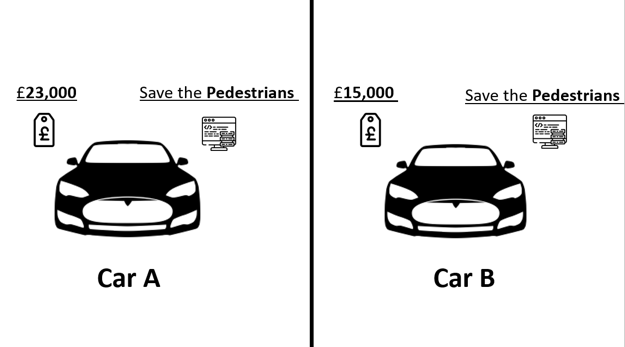
どちらの車を購入したいですか？

- A (1)
- B (2)

End of Block: 15

Start of Block: 16

Q50
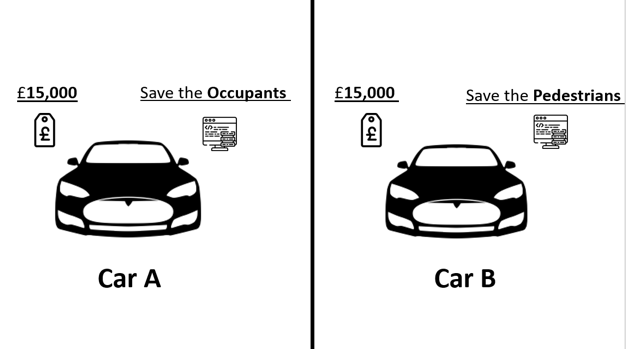
どちらの車を購入したいですか？

- A (1)
- B (2)

End of Block: 16

Start of Block: 17

Q51
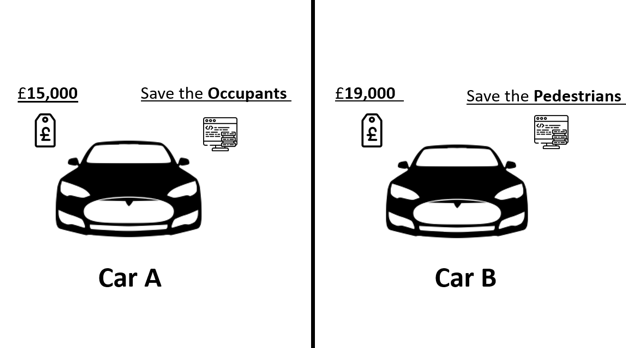
どちらの車を購入したいですか？

- A (1)
- B (2)

End of Block: 17

Start of Block: 18

Q52
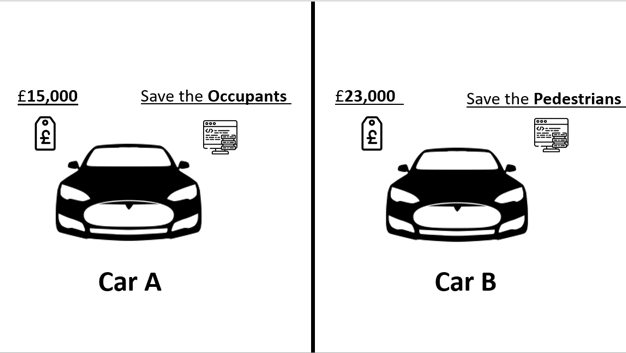
どちらの車を購入したいですか？

- A (1)
- B (2)

End of Block: 18

Start of Block: 19

Q53
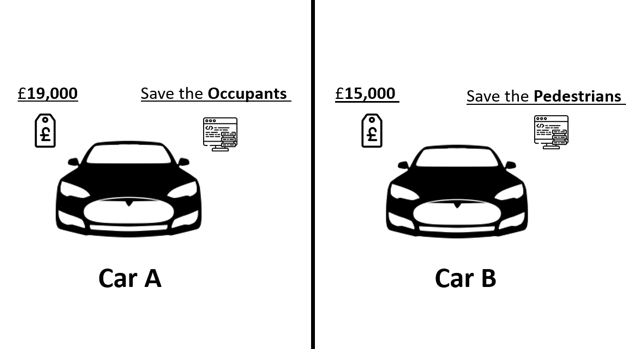
どちらの車を購入したいですか？

- A (1)
- B (2)

End of Block: 19

Start of Block: 20

Q54
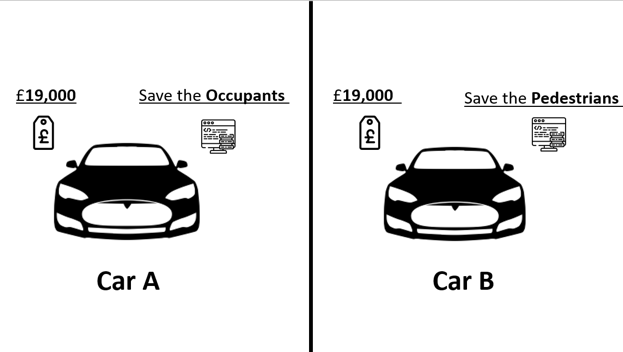
どちらの車を購入したいですか？

- A (1)
- B (2)

End of Block: 20

Start of Block: 21

Q55
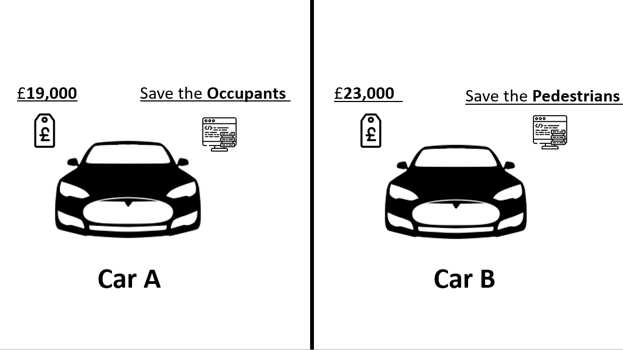
どちらの車を購入したいですか？

- A (1)
- B (2)

End of Block: 21

Start of Block: 22

Q56
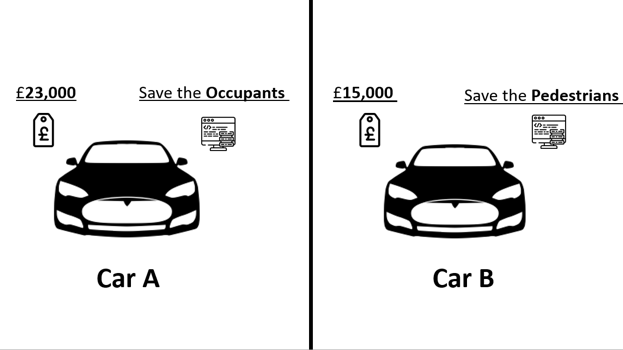
どちらの車を購入したいですか？

- A (1)
- B (2)

End of Block: 22

Start of Block: 23

Q57
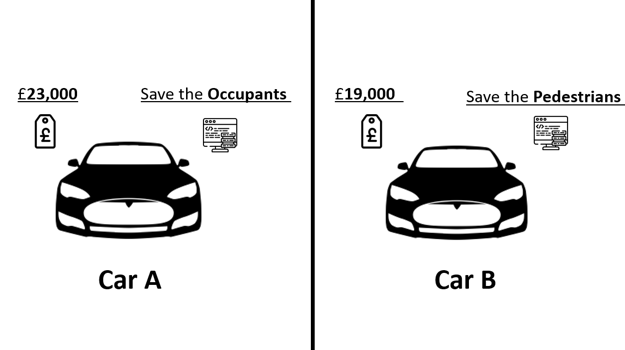
どちらの車を購入したいですか？

- A (1)
- B (2)

End of Block: 23

Start of Block: 24

Q58
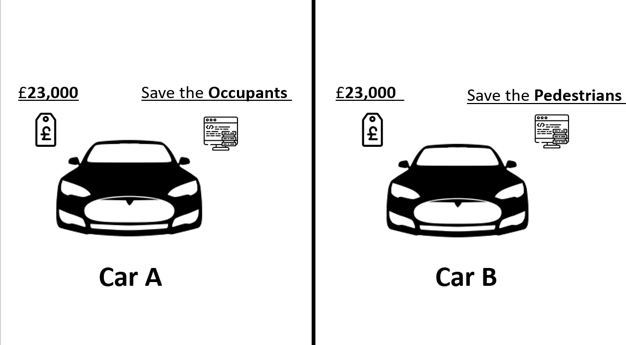
どちらの車を購入したいですか？

- A (1)
- B (2)

End of Block: 24

Start of Block: 25

Q59
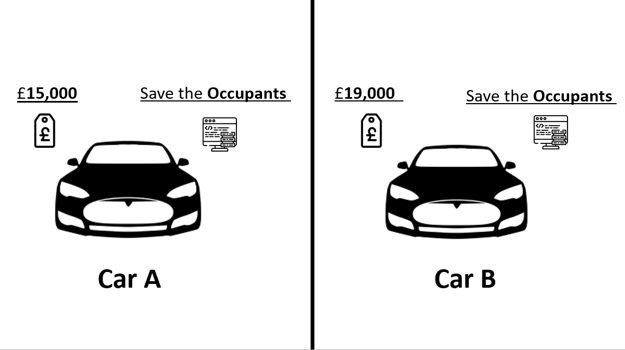
 どちらの車を購入したいですか？

- A (1)
- B (2)

End of Block: 25

Start of Block: 26

Q60
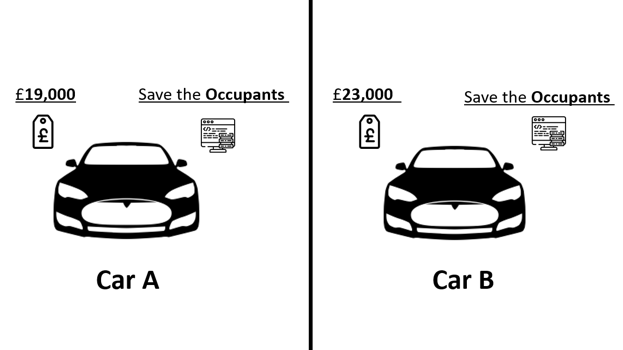
どちらの車を購入したいですか？

- A (1)
- B (2)

End of Block: 26

Start of Block: 27

Q61
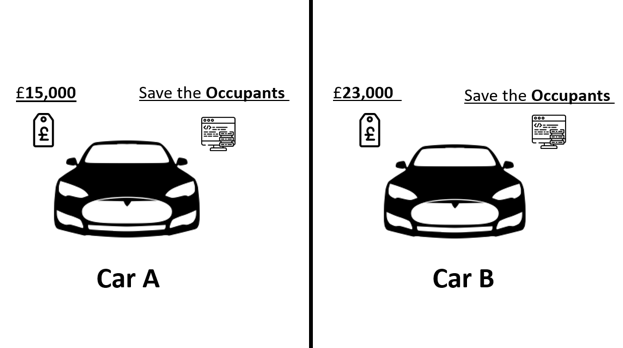
どちらの車を購入したいですか？

- A (1)
- B (2)

End of Block: 27

Start of Block: 28

Q62
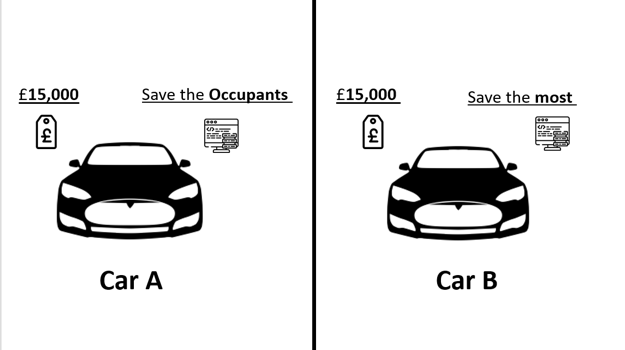
どちらの車を購入したいですか？

- A (1)
- B (2)

End of Block: 28

Start of Block: 29

Q63
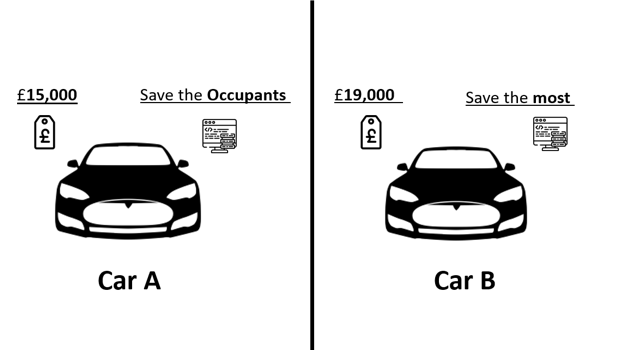
どちらの車を購入したいですか？

- A (1)
- B (2)

End of Block: 29

Start of Block: 30

Q64
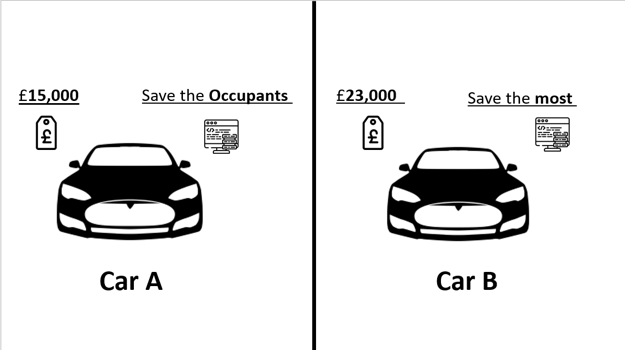
どちらの車を購入したいですか？

- A (1)
- B (2)

End of Block: 30

Start of Block: 31

Q65
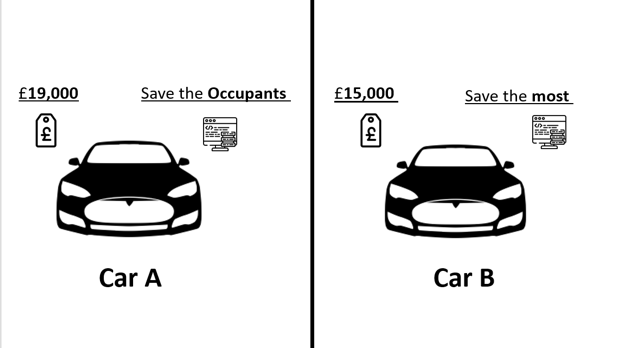
どちらの車を購入したいですか？

- A (1)
- B (2)

End of Block: 31

Start of Block: 32

Q66
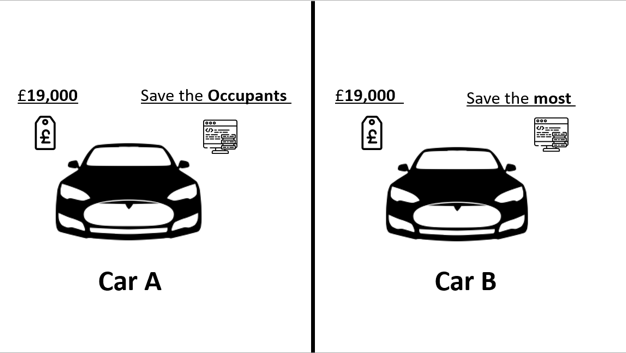
どちらの車を購入したいですか？

- A (1)
- B (2)

End of Block: 32

Start of Block: 33

Q67
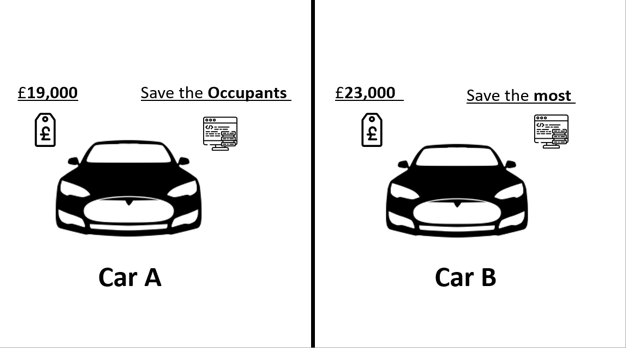
 どちらの車を購入したいですか？

- A (1)
- B (2)

End of Block: 33

Start of Block: 34

Q68
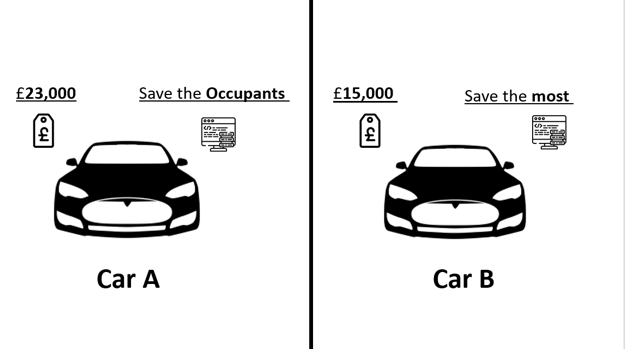
どちらの車を購入したいですか？

- A (1)
- B (2)

End of Block: 34

Start of Block: 35

Q69
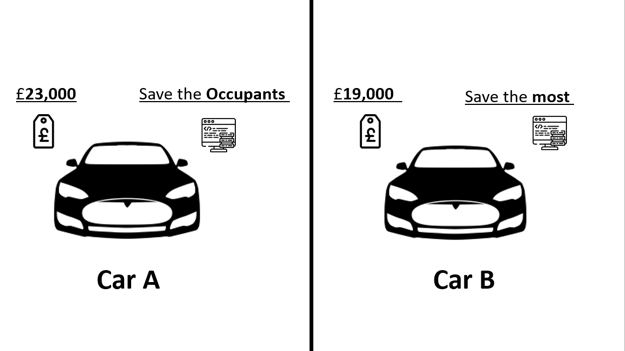
どちらの車を購入したいですか？

- A (1)
- B (2)

End of Block: 35

Start of Block: 36

Q70
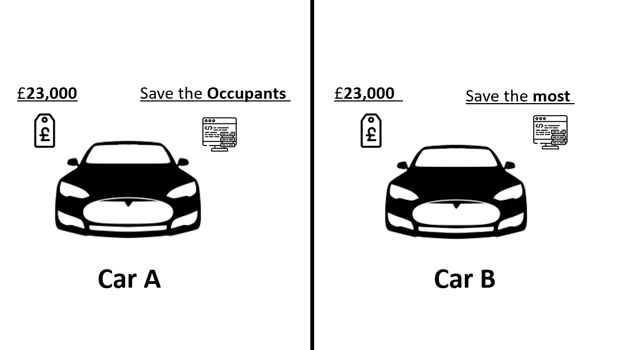
どちらの車を購入したいですか？

- A (1)
- B (2)

End of Block: 36

Start of Block: How utilitarian they are?

Q71 最後に、皆様の倫理観を測るための簡単な質問にご回答ください（9問あります）。

| Page Break |  |
| --- | --- |

Q72 Q1.　他人の命を救うためには自分の片足を犠牲にするしかない場面、その犠牲を払うことは倫理的に求められる。

- 強く反対する (1)
- 反対する (2)
- 少し反対する (3)
- どちらともいえない (4)
- 少し賛成する (5)
- 賛成する (6)
- 強く賛成する (7)

Q73 Q2.　倫理的な見方をすると、私たちは自分の腎臓の一つを腎不全の人に提供する義務がある。なぜならば、私たちは腎臓が一つあれば健康に生活できるからである。

- 強く反対する (1)
- 反対する (2)
- 少し反対する (3)
- どちらともいえない (4)
- 少し賛成する (5)
- 賛成する (7)
- 強く賛成する (13)

Q74 Q3.　倫理的な見方をすると、私たちは地球上のすべての人の幸福を平等に配慮すべきである。すなわち、私たちは個人的な感情をもとに、親しい人の幸福を優先すべきではない。

- 強く反対する (1)
- 反対する (2)
- 少し反対する (3)
- どちらともいえない (4)
- 少し賛成する (5)
- 賛成する (6)
- 強く賛成する (7)

Q75 Q4.　他人を救えるのに救わないことは、他人を積極的に傷つけるのと同じくらい悪いことである。

- 強く反対する (1)
- 反対する (2)
- 少し反対する (3)
- どちらともいえない (4)
- 少し賛成する (5)
- 賛成する (7)
- 強く賛成する (8)

Q76 Q.5　必要以上に貯金することは倫理的に間違いである。なぜならば、そのお金をより必要とする人に寄付すべきだからである。

- 強く反対する (1)
- 反対する (2)
- 少し反対する (3)
- どちらともいえない (4)
- 少し賛成する (5)
- 賛成する (6)
- 強く賛成する (7)

Q77 Q.6　多くの無実の人を救うためであれば、一人の無実の人を傷つけることは倫理的に正しい。

- 強く反対する (1)
- 反対する (2)
- 少し反対する (3)
- どちらともいえない (4)
- 少し賛成する (5)
- 賛成する (6)
- 強く賛成する (7)

Q78 Q.7　社会全体の幸福と福祉を保証する唯一の手段が政治によって市民の自由を短期間だけ抑圧することであれば、それは正当化される。

- 強く反対する (1)
- 反対する (2)
- 少し反対する (3)
- どちらともいえない (4)
- 少し賛成する (6)
- 賛成する (7)
- 強く賛成する (8)

Q79 Q.8　無実の人を拷問することで、何百人の命を危険にさらす爆弾の爆発を阻止できるのであれば、その拷問は倫理的に正当化される。

- 強く反対する (1)
- 反対する (2)
- 少し反対する (3)
- どちらともいえない (4)
- 少し賛成する (5)
- 賛成する (6)
- 強く賛成する (7)

Q80 Q.9　多くの人の命が助かるのであれば、無実の人の命が巻き添えになって犠牲になることが必要な場合もある。

- 強く反対する (2)
- 反対する (3)
- 少し反対する (4)
- どちらともいえない (5)
- 少し賛成する (6)
- 賛成する (8)
- 強く賛成する (9)

End of Block: How utilitarian they are?

Start of Block: Thank You

Q189 ご回答ありがとうございました。以下のパスコードをCrowdworksの質問に記入してください（このコードを正しくCrowdworksの二つ目の質問にご入力いただかなければ、報酬は支払われません。）
   **37640E0Z**  

End of Block: Thank You

Start of Block: Give up

Q131
正しい選択肢を選んでいただけませんでした。
この質問は無回答とさせていただきます。

次の質問へとお進みください。

End of Block: Give up
